# Long-term evaluation of a zoned catch-neuter-vaccinate-release program integrating owner engagement and buffer zone strategies for humane dog population management and rabies control in Anuradhapura, Sri Lanka

**DOI:** 10.14202/vetworld.2026.2816-2849

**Published:** 2026-07-10

**Authors:** Chamith Nanayakkara, Udaya Ayeshmantha Wijayawardana, Arundi Aparnavi Jayasekara

**Affiliations:** 1 Vets for Future, 3 /6 G, Anderson Housing Complex, Park Road, Colombo 05, Sri Lanka.

**Keywords:** animal welfare, catch-neuter-vaccinate-release, dog population management, owner engagement, rabies control, Sri Lanka, veterinary public health, zoonotic disease

## Abstract

**Background and Aim:**

Catch-neuter-vaccinate-release (CNVR) is recognized as a humane and sustainable strategy for controlling free-roaming dog populations and eliminating dog-mediated rabies. However, long-term evaluations incorporating spatial planning and community participation remain scarce. This study assessed a five-year zoned CNVR program integrating owner engagement and a peripheral buffer zone strategy in Anuradhapura, Sri Lanka, a religious heritage city affected by frequent puppy dumping and high densities of free-roaming dogs.

**Materials and Methods:**

From 2021 to 2025, sexually intact free-roaming dogs were captured along predefined routes with standardized effort, while owned dogs were voluntarily presented to clinics. The municipality was divided into three zones according to anthropogenic characteristics, and a surrounding buffer zone was progressively intensified. Population trends were analyzed using negative binomial regression models. A questionnaire survey conducted in 2025 evaluated anti-rabies vaccination (ARV) and sterilization coverage among owned dogs. Spatial and household-level factors influencing coverage were examined using generalized linear mixed models.

**Results:**

The number of free-roaming dogs requiring sterilization declined from 1,368 in 2021 to 484 in 2025. Although annual declines in community dog counts per unit effort were not statistically significant, buffer zone interventions produced significant sex- and zone-specific effects, reducing male populations in zone 1 and female populations in zone 3. Owned dog numbers increased by 14.7% annually, primarily because female ownership increased by 24.1%. More than 78% of adult female dogs were sterilized. Questionnaire responses from 1,123 participants showed that 94.2% and 83.1% of owned dogs within and outside the municipality, respectively, had received at least one ARV, while annual vaccination coverage was 74.2% and 68.9%, respectively. Household-level analyses demonstrated that geographic location, rather than the number of dogs per household, explained most of the variation in vaccination and sterilization coverage, identifying several low-coverage locations that require targeted interventions.

**Conclusion:**

A strategically zoned CNVR program incorporating owner participation and a boundary-sealing buffer zone effectively promoted humane management of the dog population and improved rabies control. Spatially targeted interventions and continued vaccination and sterilization campaigns are essential for achieving sustainable elimination of dog-mediated rabies in culturally complex urban environments.

## INTRODUCTION

Rabies is a viral zoonotic disease that is currently classified as incurable and has the highest case-fatality rate among zoonotic diseases, approaching 100% [1–5]. The number of rabies-related human deaths per annum is estimated to be 59,000 [6]. The highest death rates are reported from Asia (59.6%) and Africa (36.4%) [7], earning rabies recognition as a neglected tropical disease [8–11].

The global economic burden of rabies is estimated to be US$8.6 billion. It is worth highlighting that 80% of rabies-related human deaths occur in rural areas, and 40% of the victims are children from Asian and African regions [6, 8]. Therefore, a disproportionate burden of the disease falls on the most resource-poor countries and communities [6–17]. It is essential to note that 97% of this economic burden is attributed to loss of income resulting from premature deaths (54%), lost income while seeking treatment (15%), livestock losses (6%), travel costs (2%), and other direct costs (20%). Ironically, 37% of the economic burden is associated with post-exposure treatments (direct costs, loss of income, and travel costs), despite less than 3% of the funds being allocated to rabies eradication efforts (2% for dog vaccination and population control and only 0.01% for rabies surveillance) [6]. Poor surveillance is believed to underestimate the number of rabies cases reported regionally [6, 7, 18], making policymakers reluctant to prioritize rabies eradication in national policies [17]. For example, only one-third of rabies patients in Bangladesh are taken to hospitals, whereas the remainder die at home, resulting in substantial underreporting of rabies deaths [12]. Cambodia was reported to have had the highest number of rabies deaths per capita in 2007, yet the exact figures remained unknown even by 2024 [19]. Even when national and global interests converge, the absence of epidemiological data and poorly structured surveillance systems undermines the success of rabies eradication efforts [14]. Dog population management initiatives that are overly generic, fail to address context-specific issues, and are implemented in a scattered and poorly coordinated manner have been identified as major reasons for their failure [20]. Furthermore, these estimates do not account for indirect economic losses, such as adverse effects on tourism and threats to wildlife conservation.

However, it should be acknowledged that rabies is entirely preventable [5, 13, 21]. Owned and free-roaming dog populations are responsible for an estimated 95%–99% of human rabies cases [1, 3, 6, 10, 15, 19, 22, 23]. Mass vaccination of domestic dogs is widely accepted as the most cost-effective and humane strategy for preventing dog-mediated rabies [1, 2, 5, 12, 19, 24]. Controlling dog-mediated rabies through dog vaccination is considered more financially feasible than relying on human post-exposure prophylaxis [2]. However, the long-term sustainability of mass vaccination as the sole strategy for preventing dog-to-human transmission of rabies has been questioned in certain circumstances [1]. Although mass vaccination without sterilization has been promoted by some groups, mathematical models suggest that it may contribute to the growth of free-roaming populations by reducing juvenile mortality, thereby hindering future anti-rabies vaccination (ARV) efforts from achieving the desired vaccination coverage [25].

The United Against Rabies Collaboration, represented by the World Health Organization, the World Organization for Animal Health, the Food and Agriculture Organization of the United Nations, and the Global Alliance for Rabies Control, was established in 2015 with the goal of achieving zero human dog-mediated rabies deaths by 2030 through a One Health approach. The One Health concept proposes a three-phase strategy that prioritizes societal change through the effective use of vaccines, medicines, tools, and technologies; the generation, innovation, and measurement of impact; and sustained commitment and resource allocation. This approach promotes collaboration between the human and veterinary health sectors [6], as well as among multiple ministries and both public and private sectors. Increasing the annual budget allocation for rabies eradication from US$0 to US$7 million during 2011–2016 and to US$33 million during 2017–2022 was instrumental in reducing annual rabies incidence in Bangladesh from approximately 2,000 cases before the intervention to 200 cases in 2015. Bangladesh adopted a comprehensive multisectoral approach by training thousands of dog catchers and vaccinators, conducting three annual rounds of mass dog vaccination campaigns in every district, and strengthening surveillance systems, laboratory capacity, and human resources [12].

Addressing rabies has positive implications for food security and animal welfare while contributing to safer cities and healthier ecosystems. Rabies awareness promotes responsible pet ownership, humane dog population management, and healthier relationships between humans and dogs, thereby improving overall animal welfare [13].

Despite these global advances, long-term evaluations of context-specific dog population management programs remain limited, particularly in Sri Lanka. Existing interventions often focus on vaccination or sterilization alone, with insufficient attention to spatial zoning, boundary effects, owner participation, and sociocultural barriers that influence program sustainability. Therefore, this study aimed to evaluate a long-term, zoned catch-neuter-vaccinate-release (CNVR) program that integrates owner engagement and a peripheral buffer zone strategy for humane dog population management and rabies control in Anuradhapura, Sri Lanka.

In the Sri Lankan context, rabies has remained endemic [26] since colonial times, as evidenced by the Rabies Ordinance of 1893 [5]. Sri Lanka is one of the earliest South Asian countries to prioritize rabies control [27] and is considered to have strong potential to achieve the target of zero rabies-related deaths by 2030 [11, 21]. The establishment of the Public Health Veterinary Unit, as recommended by the World Health Organization in 1953, the declaration of human rabies as a notifiable disease in 1971, and the declaration of animal rabies as a notifiable disease in 2012 represent important milestones in the rabies eradication movement in Sri Lanka. Despite incorporating rabies into national five-year development plans by 1959 and initiating a nationwide rabies eradication program in 1975, these measures were implemented effectively only from the 1980s onward [27]. The current institutional framework, in which public funds are distributed among several ministries, has been identified as a challenge to the effective implementation of a One Health approach [2].

The practice of culling free-roaming dogs as a population control strategy, which had continued since the early 1970s, was discontinued in 2006 under the "No-Kill Policy" [21, 28] and replaced by a national sterilization program in 2008. Since then, Sri Lanka has focused primarily on dog vaccination and female sterilization in addition to administering post-exposure prophylaxis to human bite victims [5, 11].

Although Sri Lanka has achieved appreciable progress in reducing annual human rabies deaths from 300–400 during the 1970s to fewer than 30 in recent decades [29], poor vaccination coverage among owned dogs and ineffective management of free-roaming populations due to owner negligence have been cited as major factors delaying the success of the eradication program [30]. Furthermore, the absence of systematic dog population surveys, the implementation of vaccination campaigns only once per year, and inadequate allocation of human resources to regional rabies eradication activities have been identified as key limitations of the national program. The estimated expenditure on human post-exposure prophylaxis alone in Sri Lanka is approximately 700 million Sri Lankan Rupee (LKR) [27].

Dog population management can reduce problems associated with free-roaming dog populations [31], including zoonotic diseases of public health importance such as rabies, attacks on pets, livestock, and wildlife, road traffic accidents, and public nuisance, while simultaneously improving the welfare of free-roaming dogs [21]. Numerous strategies, including mass culling, sheltering, reducing carrying capacity through waste management, promoting responsible pet ownership, and surgical sterilization, have been proposed for dog population management [31, 32]. Currently, surgical sterilization coupled with ARV, popularly referred to as the CNVR approach, remains the most effective humane solution [33]. This strategy helps maintain stable vaccination coverage by reducing dog population turnover and size [34], thereby supporting the long-term achievement and maintenance of herd immunity. The World Health Organization recognizes that 70% vaccination coverage is necessary to maintain herd immunity in susceptible dog populations, regardless of population turnover [7, 35].

Real-world evaluations of the economic benefits of CNVR programs are limited both globally and within Sri Lanka. However, a comparable 22-year study conducted in Jaipur, India, demonstrated that combining animal birth control with additional vaccinations yielded a benefit-cost ratio of 8.5 when only medical costs were considered, and increased to 58.4 when broader societal benefits were included [36]. A Sri Lankan example from Colombo [2] was inconclusive about the exact benefit-cost ratios; nevertheless, it suggested that the societal benefits generated by such interventions are likely to justify the additional costs associated with planning and implementation.

Despite increasing recognition of the value of CNVR, long-term studies evaluating its effectiveness under Sri Lankan conditions remain scarce. Most available studies have been limited by short durations, restricted geographical coverage, or inadequate consideration of sociocultural factors and the movement of dogs across administrative boundaries. Consequently, there is insufficient evidence regarding the effectiveness of integrating owner participation and spatial strategies into sustainable dog population management programs. Therefore, the present study sought to address these knowledge gaps by evaluating a long-term zoned CNVR program and investigating the contribution of a peripheral buffer zone to the improvement of humane dog population management and rabies control.

The dog population control model program that formed the basis of this research was delegated to the Association of Veterinarians for Humane Management of Animal Populations (Vets for Future), the organization represented by the authors, by the Public Health Veterinary Service of the Ministry of Health in 2020 in response to a public nuisance complaint by the chief priest of Ruwanwelisaya, who requested the removal of free-roaming dogs from the temple premises. Preliminary investigations revealed that the problem was more widespread than initially anticipated, with organized networks transporting unwanted puppies from surrounding rural and suburban areas to the religious city. The concentration of temples in the northern periphery of the city center contributed to a high incidence of free-roaming dogs throughout the city. After convincing stakeholders that relocating dogs would not solve the problem in a setting characterized by abundant food resources and frequent dumping, the first author obtained permission from provincial authorities to conduct a 5-year mass sterilization-vaccination program. The surgical program was funded by Justice for Animals Sri Lanka, whereas the research itself was self-funded.

The project provided an opportunity to evaluate the feasibility of implementing a mass sterilization-vaccination campaign in a religious city surrounded by low-income neighborhoods, where the dumping of puppies at religious sites has contributed to a large free-roaming dog population. The interest shown by a religious leader in requesting a dog population management model in a high-visibility heritage city adds to the uniqueness of this study. Buddhist temples are frequently used as convenient dumping sites for unwanted litters, and this phenomenon extends even to locations of exceptional cultural importance, such as Ruwanwelisaya. In addition, the perception among some Buddhists that the sterilization of animals is sinful contributes to the initial reluctance or resistance of owners to sterilize their pets. In such a context, conducting CNVR camps within the religious city and receiving open support from Buddhist clergy were instrumental in overcoming public resistance. Although the authors could not obtain records of public nuisance complaints before the intervention because of confidentiality concerns, the Municipal Veterinary Office of the Anuradhapura Municipal Council continued to receive complaints during the intervention period (2023: 47 complaints, 2024: 38 complaints, and 2025: 51 complaints), indicating that dog population management remained a public priority.

In a setting where limited accessibility to veterinary facilities and sterilization services encourages low-income communities to abandon unwanted litters, thereby increasing the free-roaming population, sterilization of both owned and free-roaming dogs was considered equally important. The inclusion of owned dogs and the voluntary participation of community members in tracking free-roaming dogs in their neighborhoods are distinctive features of this study, as previous studies in Sri Lanka have focused primarily on stray dog populations. Furthermore, this is the only reported continuous dog population management study extending beyond 5 years, whereas most local and international studies have been limited to 3 years.

In the absence of natural geographical barriers, the influx of dogs from neighboring areas remains a major challenge that can undermine the success of CNVR programs. The establishment of a buffer zone surrounding the municipal boundary, which was intensively covered during the CNVR program, represents a novel application of the buffer zone concept commonly used in epidemiological investigations and wildlife monitoring programs to the context of dog population management.

Although the present study does not directly evaluate temporal variation in catchability, it acknowledges that catchability is likely to decline over time. Therefore, the program relied on observations made by residents, voluntary dog feeders, and a detailed photographic database maintained by the catching team to identify dogs that had escaped previous catching attempts and still required sterilization, rather than relying exclusively on conventional street surveys.

Despite growing recognition of the value of CNVR, evidence on the long-term effectiveness of systematically zoned interventions integrating owner participation and boundary-sealing strategies remains extremely limited. Previous studies in Sri Lanka have focused predominantly on stray populations and have not investigated the contribution of surrounding buffer zones in reducing immigration of free-roaming dogs into target areas. Moreover, the influence of free sterilization services on ownership patterns, age structure, and vaccination coverage among owned dogs has not been adequately studied. These knowledge gaps underscore the need for long-term evaluations that incorporate sociocultural, spatial, and demographic factors affecting sustainable rabies control.

The primary objective of this research was to investigate the contribution of this project to reducing dog populations within the Anuradhapura Municipality and its surrounding areas.

**The specific objectives were:** (a) to evaluate the overall reduction in owned and free-roaming dog populations within the Anuradhapura Municipality based on changes in the numbers of owned and community dogs recorded; (b) to determine the effect of providing free sterilization services to low-income communities on their willingness to adopt female dogs; (c) to investigate how the sterilization program influences different age groups within the population; (d) to assess the contribution of establishing a buffer zone to the reduction of free-roaming dog populations within the municipal boundary; (e) to determine ARV and sterilization coverage among owned dog populations living within the municipality and surrounding neighborhoods; (f) to evaluate the influence of keeping multiple dogs per household on the rates of regular ARV and sterilization when both services are offered free of charge; and (g) to assess secondary animal welfare benefits, including reductions in skin disorders and transmissible venereal tumor (TVT) incidence.

By addressing these objectives, the present study aimed to provide evidence for the development of sustainable, humane, and context-specific dog population management strategies that support long-term rabies control in culturally complex urban environments.

## MATERIALS AND METHODS

### Ethical approval

The surgical procedures (spaying and neutering) were performed by a team of qualified veterinarians registered with the Sri Lankan Veterinary Council. Since only routine procedures were performed and no experimental interventions involving live animals were undertaken, formal ethical approval for the surgeries was not required. Owned animals were sterilized only after obtaining the owners’ consent. Free-roaming animals were sterilized with the approval and supervision of the relevant local government authorities, namely the Anuradhapura Municipal Council and the Manupa, Nanupa, and Mihinthale Pradeshiya Sabhas. Free-roaming animals were captured, vaccinated, sterilized, and returned to their original habitats after adequate recovery in accordance with internationally recognized CNVR protocols [37] and the field clinic guidelines of the Department of Animal Production and Health. Animals that were sick, pregnant, lactating, very young, or very old were excluded from sterilization.

The questionnaire survey, which was conducted to determine the proportion of pets that were sterilized and vaccinated against rabies, was preceded by a detailed verbal explanation to pet owners regarding the importance of the survey, and written informed consent was obtained through signed response forms. Prior written permission to conduct the survey was obtained from the Provincial Commissioner of North Central Province. The survey was conducted in accordance with the principles of the Declaration of Helsinki.

### Study period and location

**Figure 1:**
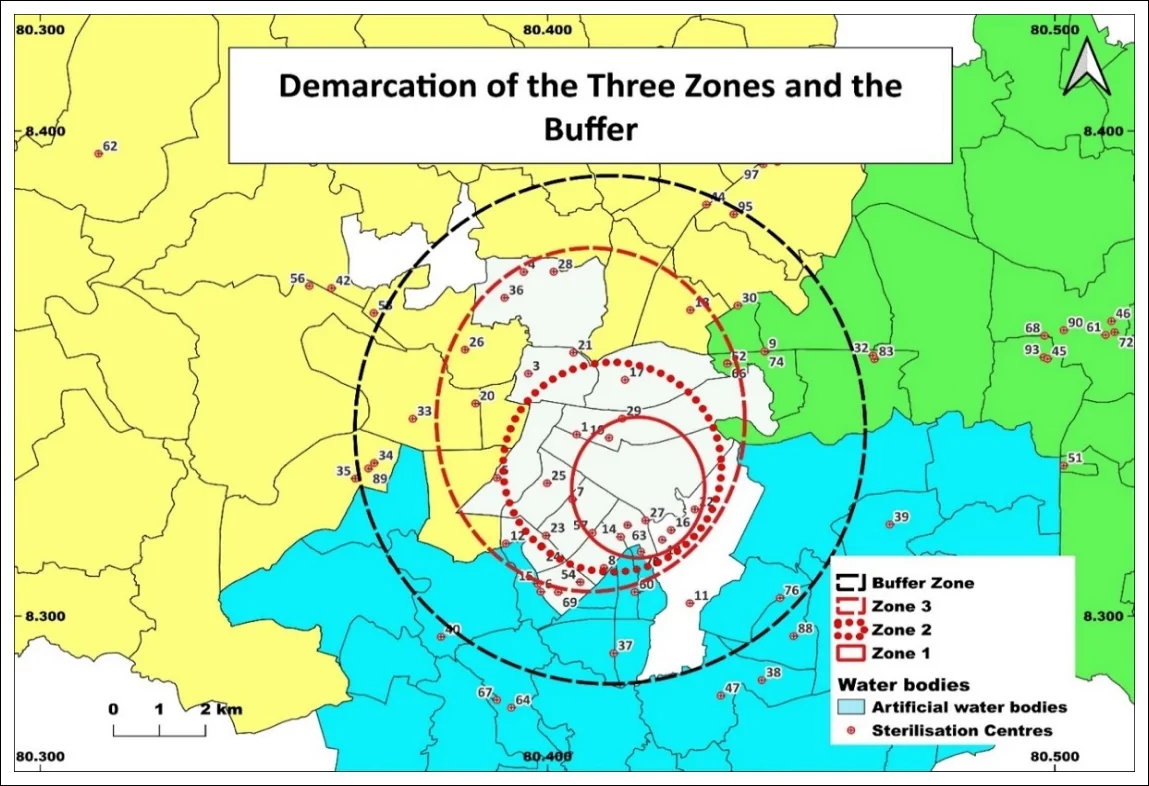
Distribution of sterilization centers and demarcation of zonal boundaries within the Anuradhapura Municipality from 2021 to 2025.

The questionnaire survey was conducted from September to October 2025. The dog population control model program was initiated in May 2020 at the Municipal Veterinary Office of Anuradhapura, with the initial activities concentrated in the ancient city, which is characterized by a high density of free-roaming dogs. Based on observations made during 2020, the CNVR program adopted a more systematic approach beginning in March 2021. The Anuradhapura Municipality was divided into three circular zones according to demographic characteristics (Figure 1).

The first zone (8°20′26.01″N–8°18′29.03″N and 80°24′17.55″E–80°25′21.81″E) had a diameter of 2.6–2.9 km and an area of 5.94 km², encompassing the commercial city, consisting mainly of shops, offices, and transport hubs. The dog population in this region consisted predominantly of strays (free-roaming dogs without owners or responsible caregivers), representing the less catchable fraction.

The second zone, with a diameter of 4.3 km and an area of 8.58 km², was located between 8°21′10.68″N and 8°18′45.37″N and 80°23′23.64″E and 80°25′59.79″E and surrounded Zone 1. This zone primarily covered the ancient city and included only a limited number of residential areas, where dumping of unwanted puppies was frequently observed.

The third zone, covering 18.66 km² with a horizontal diameter of 6 km and a vertical diameter of 7 km, extended between 8°22′54.77″N and 8°18′9.05″N and 80°23′7.59″E and 80°26′21.71″E. This zone surrounded the first two zones and consisted mainly of residential areas. The dog population comprised owned and community dogs (dogs without a single owner but collectively cared for by community members), the majority of which were free-roaming. The boundary of this zone, represented by a red dashed line, approximated the municipal boundary of Anuradhapura. The total area of the municipality is 36.32 km², and the three circular zones collectively covered 33.18 km², corresponding to 91.35% coverage. The peripheral areas excluded from the third circle were included in the buffer zone.

Owners brought their dogs to the clinics for sterilization, whereas teams of dog catchers captured free-roaming stray and community dogs for ARV and sterilization. Both male and female dogs that had reached puberty were selected for sterilization without intentional sex bias. In contrast, owners tended to prioritize the sterilization of female animals.

The geographic coordinates of sterilization centers and catching locations were recorded using Google Earth Pro (±5 m accuracy), and all maps were developed using QGIS version 3.44.5.

During 2021 and 2022, sterilization centers and catching activities were confined to the three zones within the Anuradhapura Municipal Council boundaries. However, no marked reduction in the number of unsterilized free-roaming dogs or the dumping of new litters was observed by the end of 2022, highlighting the necessity of "sealing the boundary to cut down the supply." Consequently, the project boundary was extended by 3 km beyond the municipal boundary, resulting in a circle with a diameter of 10 km and an area of 45.36 km², excluding the first three zones. This area was designated as the buffer zone, represented by a black dashed line in Figure 1. The buffer zone was prioritized during 2024 and 2025 to minimize the influx of intact adult dogs and the dumping of puppies into Zones 2 and 3.

The zonal demarcation relied heavily on anthropogenic features such as commercial districts and residential areas, which are known to influence dog behavior [38]. Furthermore, municipal boundaries had to be considered because of state supervision. The actual land area of each zone was smaller than the calculated values due to the presence of large water reservoirs. The 10-km-diameter buffer zone (3 km beyond the municipal boundary) was selected because its anthropogenic characteristics and human population density are similar to those of Zone 3. Areas beyond 3 km are more sparsely populated and have a lower density of free-roaming dogs. Moreover, the roaming distance of Sri Lankan dogs rarely exceeds 2 km, making a 3-km buffer sufficient to minimize immigration into the city. Edge effects were not considered in the calculations.

The Anuradhapura Municipality is surrounded by three Pradeshiya Sabhas (local government areas), namely Nuwaragam Palatha Central (Manupa), Nuwaragam Palatha East (Nanupa), and Mihinthale. Regions within and beyond the buffer zone belong to one of these administrative divisions. Project activities were extended to these neighboring areas by 2022 as an additional precaution against the influx of dogs into the city.

**Figure 2:**
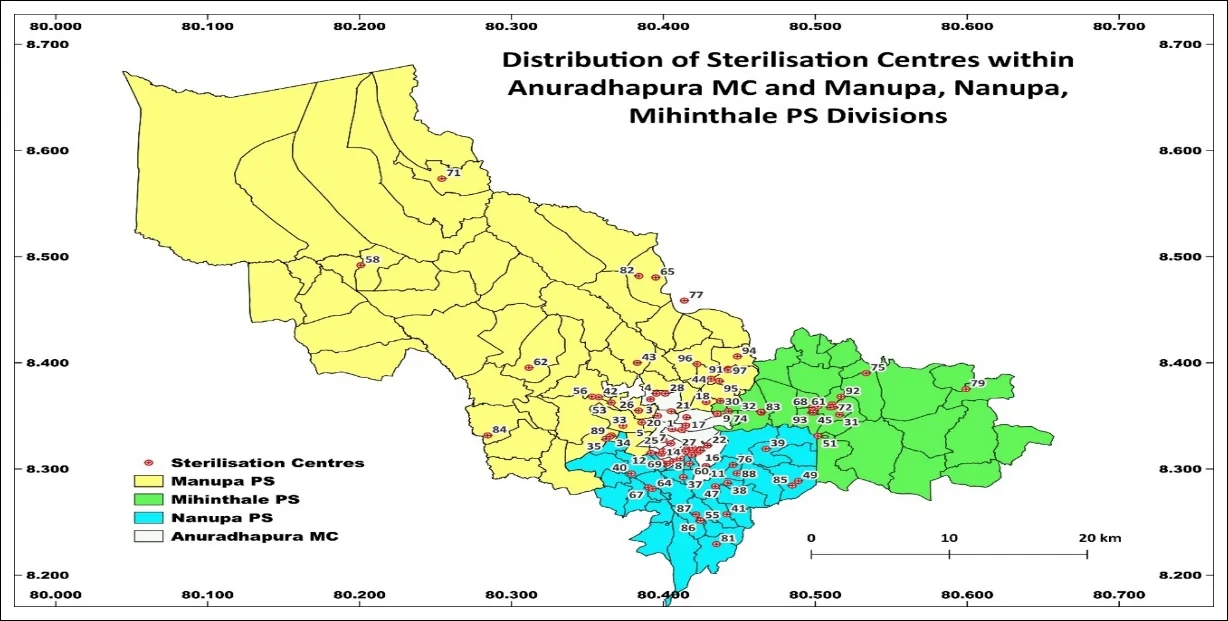
Distribution of the sterilization centers within the Manupa, Nanupa, and Mihinthale Pradeshiya Sabha boundaries.

Figure 2 illustrates the distribution of sterilization centers within the boundaries of the three Pradeshiya Sabhas and the Anuradhapura Municipality. Manupa is shown in yellow, Nanupa in cyan, and Mihinthale in pale green.

Table 1A summarizes the time frame and geographical coverage of the sterilization programs. Table 1B presents the numbers of anti-rabies vaccinations and sterilizations performed by the Municipal Veterinary Office from 2014 to 2019 for comparison.

The human population of the Anuradhapura Municipality is currently 63,276, and based on the national dog-to-human ratio of 1:8, the estimated dog population is approximately 7,910.

Conducting at least four sterilization programs annually, with intervals not exceeding 3 months, considerably reduced the probability of females that had escaped sterilization during previous campaigns becoming pregnant, because the reproductive cycle of dogs in Sri Lanka typically lasts approximately 6 months.

### Outreach strategy and catching process

Areas with a high density of stray and free-roaming dogs within the municipality were identified during preliminary surveys conducted in 2020. Collaboration with voluntary dog feeders facilitated the identification of locations requiring special intervention.

**Table 1A:** Summary of the time frame and the sterilization coverage of the project.

**Year**	**The number of mass sterilization** **programs conducted per year**	**Total number of catching days per year**	**Zones covered**	**Anti rabies** **vaccination for dogs**	**Number of sterilizations**
2020*	2	8	Preliminary events before the demarcation of the zones*	777	Male dogs: 94
					Female dogs: 575
					All dogs: 669
2021	6	54	1,2,3	5742	Male dogs: 927
					Female dogs: 1789
					All dogs: 2716
2022	6	47	1,2,3, Buffer, Manupa, Nanupa, Mihinthale	8128	Male dogs: 765
					Female dogs: 1862
					All dogs: 2627
2023	3	40	1,2,3, Buffer, Manupa, Nanupa, Mihinthale	6457	Male dogs: 487
					Female dogs: 1320
					All dogs: 1807
2024	4	39	1,2,3, Buffer, Manupa, Nanupa, Mihinthale	5334	Male dogs: 266
					Female dogs: 1110
					All dogs:1376
2025	3	36	1,2,3, Buffer, Manupa, Nanupa, Mihinthale	5528	Male dogs: 248
					Female dogs: 891
					All dogs: 1139
Total					Male dogs: 2787
					Female dogs: 7547
					All dogs: 10,334

*The findings from 2020 were excluded from the statistical analyses due to limited geographical coverage and the preliminary nature of the project in 2020.

**Table 1B:** The number of sterilizations and anti-rabies vaccination (ARV) conducted within Anuradhapura MC from 2014 to 2019.

**Year**	**ARV**	**Sterilizations**
2014	3609	1467
2015	3673	1810
2016	4578	1159
2017	2924	1401
2018	2654	1509
2019	3206	1633

Two dog-catching teams, each comprising three members, were assigned two predetermined routes for each zone throughout the study period, regardless of the sterilization center's location, thereby ensuring that no streets were omitted during any campaign. Repeated coverage of the same areas ensured that dogs missed during previous campaigns, including young puppies, pregnant or lactating females, sick animals, and dogs that had escaped capture, could be sterilized during subsequent visits before reproducing and contributing to population growth.

Each route was covered during three 1.5-h shifts. The first shift commenced at 05:00 h before sunrise, when heat stress was minimal and dogs were less active and easier to capture. The second shift started at approximately 13:00 h, coinciding with the feeding activities of volunteer dog feeders, when community dogs tended to congregate at specific locations to await food. The third shift began at 16:30 h, when dogs were commonly observed following office workers and students and congregating around bus stops and shops, making them easier to capture.

The number of catching days allocated to each zone depended on necessity. Repeat visits were conducted whenever large numbers of sexually intact, free-roaming dogs of reproductive age or dogs that escaped capture were encountered. Nevertheless, both teams focused on only one zone per day, and the number of dogs captured and the duration of catching efforts remained standardized. Table 2 summarizes the number of days allocated to each zone annually.

**Table 2:** Numbers of free-roaming dogs captured per zone from 2021 to 2025.

**Year**	**Zone**	**Program no.**	**Total**	**Male**	**Female**	**No. of days**
2021	1	1	35	17	18	1
2021	1	2	139	28	111	4
2021	1	3	84	26	58	3
2021	1	4	143	37	106	3
2021	1	5	51	13	38	2
2021	1	6	77	21	56	2
2021	1	Sub total	529	142	387	15
2021	2	1	63	18	45	2
2021	2	2	66	13	53	2
2021	2	3	154	28	126	4
2021	2	4	92	23	69	2
2021	2	5	26	3	23	1
2021	2	6	99	24	75	2
2021	2	Sub total	500	109	391	13
2021	3	1	224	55	169	6
2021	3	2	160	34	126	4
2021	3	3	104	27	77	3
2021	3	4	85	26	59	4
2021	3	5	46	11	35	2
2021	3	6	146	35	111	6
2021	3	Sub total	765	188	577	25
2021	Buffer	1	24	11	13	1
2021	Buffer	2	0	0	0	0
2021	Buffer	3	0	0	0	0
2021	Buffer	4	0	0	0	0
2021	Buffer	5	0	0	0	0
2021	Buffer	6	0	0	0	0
2021	Buffer	Sub total	24	11	13	1
2022	1	1	99	27	72	2
2022	1	2	0	0	0	0
2022	1	3	14	3	11	1
2022	1	4	68	24	44	2
2022	1	5	56	18	38	2
2022	1	Sub total	237	72	165	7
2022	2	1	42	18	24	1
2022	2	2	0	0	0	0
2022	2	3	36	13	23	1
2022	2	4	89	28	61	3
2022	2	5	53	13	40	1
2022	2	Sub total	220	72	148	6
2022	3	1	157	43	114	4
2022	3	2	212	79	133	4
2022	3	3	53	16	37	2
2022	3	4	32	9	23	2
2022	3	5	0	0	0	0
2022	3	Sub total	454	147	307	12
2022	Buffer	1	0	0	0	0
2022	Buffer	2	76	26	50	1
2022	Buffer	3	129	56	73	4
2022	Buffer	4	14	4	10	1
2022	Buffer	5	4	0	4	1
2022	Buffer	Sub total	223	86	137	7
2023	1	1	49	8	41	1
2023	1	2	60	9	51	2
2023	1	3	52	15	37	2
2023	1		161	32	129	5
2023	2	1	71	19	52	2
2023	2	2	74	28	46	2
2023	2	3	64	21	43	2
2023	2	Sub total	209	68	141	6
2023	3	1	95	30	65	3
2023	3	2	62	11	47	2
2023	3	3	124	43	81	4
2023	3	Sub total	281	84	193	9
2023	Buffer	1	0	0	0	0
2023	Buffer	2	63	18	45	2
2023	Buffer	3	37	15	22	1
2023	Buffer	Sub total	100	33	67	3
2024	1	1	62	16	46	2
2024	1	2	45	17	28	1
2024	1	3	54	20	34	2
2024	1	Sub total	161	53	108	5
2024	2	1	78	18	60	2
2024	2	2	37	7	30	1
2024	2	3	39	18	21	2
2024	2	Sub total	154	43	111	5
2024	3	1	120	49	87	4
2024	3	2	81	12	69	2
2024	3	3	61	16	45	2
2024	3	Sub total	262	77	201	8
2024	Buffer	1	51	17	34	1
2024	Buffer	2	0	0	0	0
2024	Buffer	3	62	14	48	1
2024	Buffer	Sub total	113	31	82	2
2025	1	1	25	7	18	1
2025	1	2	65	21	44	1
2025	1	3	66	14	52	2
2025	1	Sub total	156	42	114	4
2025	2	1	50	7	43	1
2025	2	2	65	22	43	1
2025	2	3	21	9	12	1
2025	2	Sub total	136	38	98	3
2025	3	1	53	17	36	3
2025	3	2	73	23	50	2
2025	3	3	51	23	28	3
2025	3	Sub total	177	63	114	8
2025	Buffer	1	144	33	111	3
2025	Buffer	2	0	0	0	0
2025	Buffer	3	178	40	138	3
2025	Buffer	Sub total	322	73	249	6

Nylon nets mounted on stainless steel or aluminum frames were used for capturing dogs. The metallic frame had an internal diameter of 60 cm, the handle was 90 cm long, and the nylon net was 60 cm deep.

Young puppies and friendly dogs were captured manually. Catching vehicles moved slowly along predetermined routes while strictly adhering to zonal boundaries. Catchers covered narrow side streets on foot and followed the designated routes using Google Maps. Although this approach differed from conventional street surveys, its objective was to maximize capture efficiency and optimize resource utilization.

Neighboring communities were informed about upcoming clinics at least 1 week in advance through public announcements, handbills, and social media campaigns to ensure that owners who required the service did not miss the clinics due to lack of awareness or inadequate preparation. Announcements were delivered in Sinhala using loudspeakers along the intended routes, and more than 1,000 handbills were distributed before each campaign.

### Data collection and record keeping

**The following data were recorded for animals sterilized and vaccinated from 2020 onward:** date, location, total number of dogs, total numbers of male and female dogs, numbers of owned and free-roaming dogs, numbers of male and female dogs within the owned and community dog groups, total number of cats, numbers of male and female cats, and number of ARVs administered. In addition, dogs were classified into age groups as newborn puppies with mothers, young puppies <3 months, puppies aged 3 months–1 year, sexually active dogs aged 1–6 years, and senior dogs >6 years. Dental aging techniques were used to determine age groups. Dogs found roaming on streets without an accompanying owner or visible sign of ownership, such as a collar, were initially classified as free-roaming but reclassified as owned if ownership was later confirmed.

The questionnaire survey was designed to collect information on the number of cats and dogs living in households in different regions within and outside the municipal boundary, including their age, sex, vaccination history, and sterilization status by the end of the program. Responses included information on pets brought to 49 vaccination/sterilization centers from late September to mid-October 2025, as well as other pets in the same households that were not brought to the clinics during this period. The questionnaire used for the survey is provided as Supplementary Material 1.

The survey targeted pet owners and voluntary caregivers within the Anuradhapura Municipality and surrounding Pradeshiya Sabhas covered by the project, using convenience sampling. Cochran’s formula was used to determine the minimum required sample size, with a Z-score of 1.96 for a 95% confidence interval (CI), p = 0.5 to represent maximum variability, and e = 0.05 to represent a 5% margin of error:

n = Z²p(1 − p)/e²

The total population size of the municipality and three Pradeshiya Sabhas exceeded 100,000, resulting in a minimum required sample size of 384. For the total population of 277,812 across the four divisions (Anuradhapura Municipal Council: 60,237; Manupa: 103,641; Nanupa: 70,238; and Mihinthale: 40,657), the final survey sample of 1123 was considered sufficiently representative, with an estimated 3% margin of error. Of the 1123 responses, 737 were obtained from 41 locations within the municipality and 386 from eight locations outside the municipal boundary, among 1687 pet owners who visited the clinics. The questionnaire response rate was 66.57%.

Before implementation, the questionnaire was reviewed internally for clarity and piloted among 10 team members. Responses from the pilot survey were excluded from the analysis. The authors acknowledge the sampling bias associated with surveying pet owners who visited clinics rather than conducting a door-to-door survey, because respondents were likely to represent the more responsible segment of pet owners.

Regular ARV was defined as uninterrupted annual rabies vaccination for ≥3years. Vaccination histories were verified from vaccination records provided by pet owners.

Although the survey included cat owners, data on cats were excluded from the present analysis because cat counts were limited compared with dog counts, no homeless cats were captured, and dogs were the primary focus of the project. Therefore, households with zero dogs, including households with cats only, were excluded from the analysis.

### Statistical analysis

All statistical analyses were performed using RStudio version 4.4.2 (R Core Team, Vienna, Austria).

**Temporal trends in dog counts:** Temporal trends in counts per unit effort were quantified for nine demographic categories: total dog count; males; females; owned dogs; community dogs (free-roaming dogs cared for by the community without a specific owner); owned males; owned females; community males; and community females. Negative binomial regression was used for each category. The model count ~ Year + offset(log[number of days]) was fitted to annual totals for 2021–2025 (n = 5 years), where the offset adjusted for annual variation in the number of program days. The exponentiated Year coefficient was interpreted as the incidence rate ratio (IRR), representing the annual proportional change in catch per catching day. Each model was fitted using the glm.nb function in R v4.x. Model accuracy was assessed using the dispersion parameter θ, residual deviance/df, Pearson χ²/df, and DHARMa-scaled residuals, with consideration of uniformity, overdispersion, and zero-inflation. Influential observations were identified as Cook’s distance >0.8 (4/n = 4/5). All tests used α = 0.05.

**Figure 3:**
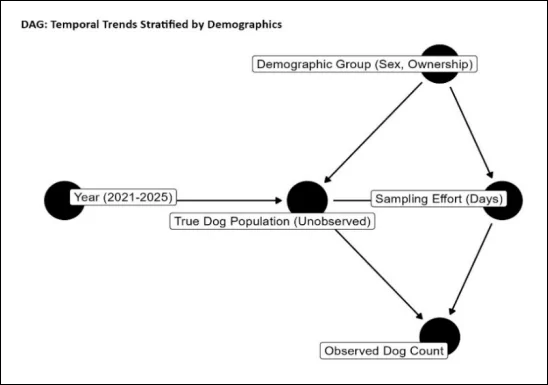
Directed acyclic graph showing temporal trends in dog populations stratified by demographic category.

A directed acyclic graph (DAG) was developed to justify the structure of the negative binomial regressions. The DAG identifies Year as the primary exposure and observed dog count as the outcome, with sampling effort acting as an endogenous variable driven by the underlying true population density. Including log(number of days) as an offset in each model appropriately blocks the confounding pathway associated with non-random sampling effort. Rather than adjusting for demographic categories, including sex and ownership status, as covariates in a single pooled model, analyses were stratified into nine distinct demographic categories. As illustrated in Figure 3, these demographic categories determine baseline population densities and are likely to influence catching effort. Stratification by demographic category prevents pooling bias and allows detection of effect modification, enabling calculation of distinct temporal IRRs for subsets such as community and owned dogs.

**Sex ratios: Annual male:female ratios were calculated for three populations:** all dogs, owned dogs, and community dogs, to determine whether sex composition changed over time. Ratios were calculated as male dogs/female dogs, owned males/owned females, and community males/community females. Community ratios were set as missing when the denominator was zero. Ordinary least squares regression with year as the predictor was used to test temporal trends in ratios (ratio ~ year). Models were fitted separately for each category. Trends were visualized by plotting ratios against year with fitted regression lines. Analyses were conducted in R 4.4.3 using dplyr, tidyr, and ggplot2.

**Figure 4:**
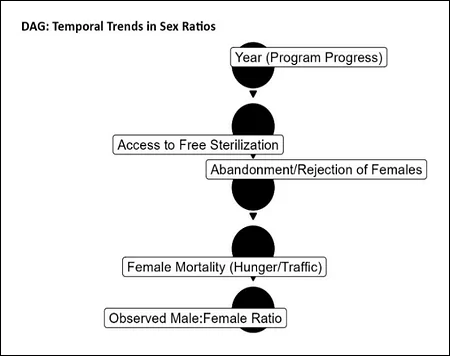
Directed acyclic graph showing the mechanistic pathway underlying changes in sex ratios.

A DAG mapping the hypothesized underlying mechanisms (Figure 4) was developed to justify the use of unadjusted ordinary least squares regression (ratio ~ year). Improved access to free animal birth control was hypothesized to reduce the sociological tendency to abandon or reject female dogs over time. This reduction in abandonment may subsequently reduce female mortality from factors such as starvation and traffic accidents, ultimately altering the observed male:female ratio. These sociological and mortality factors act as mediators on the causal pathway between the intervention, proxied by time, and the outcome. Adjusting for these factors would introduce overadjustment bias and block the total effect. Therefore, the DAG justifies the unadjusted ordinary least squares model as the appropriate specification for capturing cumulative temporal change in sex composition.

**Age-specific variation in dog counts:** Age-specific variation in dogs recorded per day for each year was analyzed using negative binomial generalized linear models (GLMs) with a log link and log(number of days) as an offset, thereby accounting for variation in the number of program days each year. Separate models were fitted for four age categories: <3 months, 3 months–1 year, 1–6 years, and >6 years. Year was coded as 0 for 2021 through 4 for 2025, so that exponentiated coefficients represented annual IRRs. An IRR >1 indicated an annual increase in capture rate per day, whereas an IRR <1 indicated an annual decline. IRRs are reported with 95% CIs and p-values from Wald tests. Model fit was assessed using residual deviance divided by residual degrees of freedom, with values close to 1 indicating adequate fit. Overdispersion and zero-inflation were assessed using simulation-based diagnostics in the DHARMa package. No age-specific model showed evidence of overdispersion (all dispersion test p > 0.4) or zero-inflation (all p > 0.4). Influential observations were assessed using Cook’s distance; one to two years were influential for each model, as expected with n = 5 data points. All analyses were conducted in R v4.x using MASS, broom, and DHARMa. Significance was set at α = 0.05.

**Figure 5:**
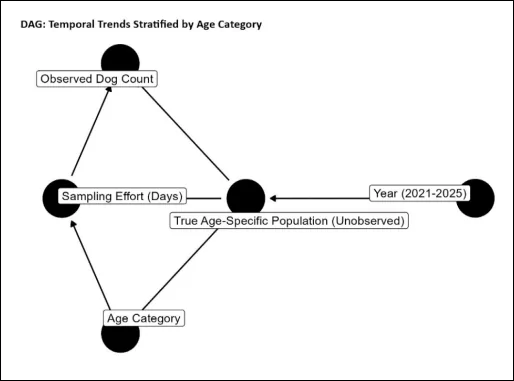
Directed acyclic graph showing temporal trends by age group.

A DAG mapping the operational and biological realities of the intervention (Figure 5) was constructed to justify the age-stratified negative binomial regression models. Field protocols prioritized adult dogs for sterilization over young puppies, indicating that age category directly influenced sampling effort. In addition, the biological mechanism of the sterilization program, namely prevention of new births, disproportionately affects the true population density of younger age cohorts compared with older cohorts and therefore acts as an effect modifier. Pooling all age groups into a single adjusted model would obscure these distinct dynamics because age category influences both operational effort and the biological outcome. The DAG explicitly justifies fitting separate models for each age group and using log(number of days) as an offset to account for sampling effort, thereby allowing estimation of the unique annual IRR for each age cohort.

**Influence of the buffer zone:** To assess the influence of introducing the buffer zone on dog counts in the first three zones, dog count data were analyzed using negative binomial GLMs to account for overdispersion, with log(number of days) included as an offset. Analyses were restricted to programs that covered the zones for at least 1 day. Because programs in the early years were mostly limited to the inner zones and excluded the buffer, two periods were defined: pre-buffer intensification (2021–2022) and post-buffer intensification (2023–2025), with 2023 marking the start of intensified buffer operations. Contemporaneous buffer spillover effects in the post-2023 period were tested using the model Total ~ Zone × Period + Zone:Period:Buffer Total + offset(log[number of days]). The three-way interaction estimated the log change in zone-specific catch per additional dog sterilized from the buffer during the same program.

To test for sex-biased responses, identical models were fitted separately for male and female counts. Before/after changes were assessed using Male ~ Zone + Zone:Period + offset(log[number of days]) and Female ~ Zone + Zone:Period + offset(log[number of days]). Buffer spillover was assessed using Male ~ Zone × Period + Zone:Period:Buffer Total + offset(log[number of days]) and Female ~ Zone × Period + Zone:Period:Buffer Total + offset(log[number of days]). Buffer Total was calculated as the sum of male and female dogs sterilized from the buffer per program.

To test the hypothesis that buffer operations triggered delayed displacement into Zone 1, lagged buffer variables were created. Buffer_Run_Lag1 was coded as 1 for Zone 1 programs immediately following a buffer program, and Buffer_Gap_Lag1 represented the number of days between a buffer program and the next Zone 1 program. The models Total ~ Buffer_Run_Lag1 + offset(log[number of days]) and Total ~ Buffer_Gap_Lag1 + offset(log[number of days]) were fitted using post-2023 Zone 1 data only. The same models were fitted separately for male and female counts.

The operational dataset constrained the sample size to nine post-2023 programs per zone. Post hoc power analysis using the pwr package indicated 80% power to detect a ≥35% change in Zone 1 catch rates, given the observed dispersion parameter θ = 11.8–18.0. For buffer spillover, 80% power was available to detect IRR ≤0.992 or ≥1.008 per buffer dog, equivalent to a ≥0.8% change per dog. Thus, significant effects of IRR = 0.9943–0.9955 represented the minimum detectable effect sizes. The models were not controlled for seasonality or climate because of limited program-level metadata. Buffer Total treated all buffer removals, defined as removal of sexually active animals from the reproducing population through sterilization and not relocation, as equivalent, although spatial locations within the buffer may have varied. Model selection was based on the Akaike information criterion. Significance was assessed at α = 0.05. All analyses were performed in R 4.4.3 using MASS for negative binomial GLMs.

**Figure 6:**
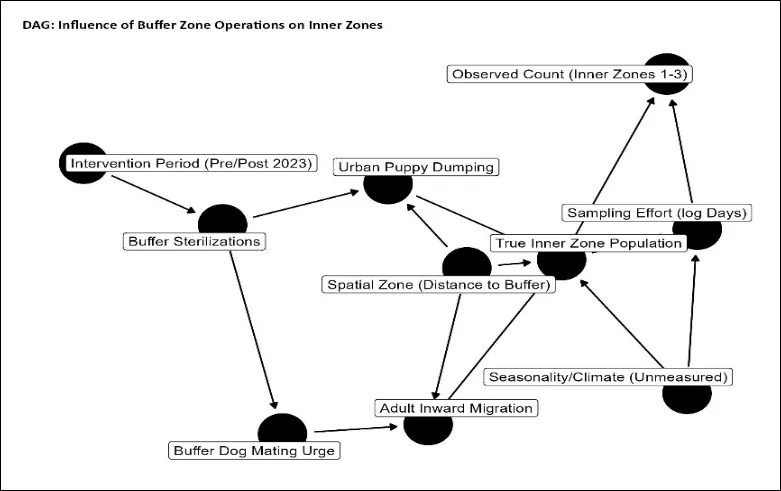
Directed acyclic graph showing the influence of the buffer zone.

A comprehensive DAG (Figure 6) was constructed to justify the spatiotemporal regression models, particularly the inclusion of the Zone:Period:Buffer Total interaction, and to address limitations arising from unmeasured variables. Intensified buffer operations after 2023 were hypothesized to reduce inner-zone dog counts through two behavioral and sociological mediators: (1) reducing the availability of unwanted puppies, thereby decreasing urban dumping by pet owners, and (2) removing the mating drive among free-roaming buffer dogs, thereby preventing temporary inward migration. The DAG further illustrates that zones serve as spatial modifiers of these mediators: Zone 3 immediately borders the buffer, whereas Zone 1 is relatively insulated. Therefore, the three-way interaction captures the expected distance-decay effect of buffer spillover. Seasonality was included as an unmeasured latent node in the DAG to account for its potential role as a confounder affecting both the true population density and operational catching effort.

**Determinants of vaccination and sterilization coverage:** Initially, crude vaccination and sterilization percentages were calculated for each of the 49 locations by dividing the total number of vaccinated and sterilized dogs by the total number of dogs per location. Negative binomial regression models were then fitted to counts of vaccinated and sterilized dogs, using log(total dogs) as an offset and mean dogs per household as the primary exposure to explore predictors of location-level coverage. IRRs with 95% CIs were reported. Model fit was assessed using R² from equivalent linear models of rates.

Recognizing that aggregate analysis can obscure within-location heterogeneity and is vulnerable to ecological bias, the data were refitted using binomial generalized linear mixed models (GLMMs), with household as the unit of analysis. For vaccination, the outcome was cbind(Annually Vaccinated, Not Vaccinated), and for sterilization, the outcome was cbind (Total Sterilized, Not Sterilized). Location No was included as a random intercept to account for clustering. From each model, the median odds ratio (MOR) was extracted to quantify between-location heterogeneity, and the intraclass correlation coefficient (ICC) was extracted to quantify the proportion of total variance attributable to location.

Location-specific adjusted probabilities were computed by adding each random intercept to the fixed intercept and back-transforming from the logit scale to identify priority locations. The 95% CIs were calculated for each location using the conditional variance of the random effects. Locations were ranked by adjusted sterilization probability. Priority locations were defined as those with an adjusted sterilization probability <35% and a 95% upper CI <50%. All analyses were conducted in R 4.3.2 using MASS, lme4, and merTools.

**Figure 7:**
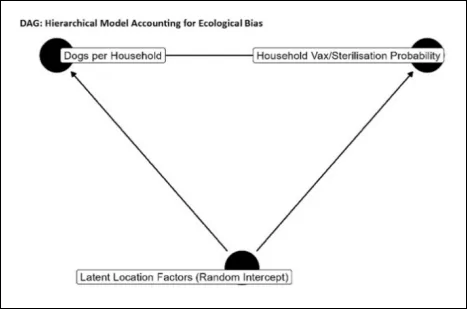
Directed acyclic graph showing determinants of vaccination and sterilization coverage.

A hierarchical DAG was constructed to justify the transition from aggregate ecological analysis to GLMMs. The DAG (Figure 7) illustrates that unmeasured location-specific characteristics, referred to as latent location factors, likely act as confounders by simultaneously influencing household dog density and the probability of vaccination or sterilization. Aggregate analysis obscures these pathways and increases the risk of ecological bias. Using a binomial GLMM with Location No. as a random intercept effectively proxies these unmeasured spatial confounders. This hierarchical structure allows the model to partition variance, quantified using ICC and MOR, block confounding pathways, and calculate adjusted location-specific intervention probabilities free from aggregation bias.

**Temporal patterns in TVT and skin treatments:** Negative binomial GLMs, with the log of mobile clinic days as an offset, were used to test temporal trends in canine TVT and skin treatment cases reported in the municipality. This approach accounted for differences in the number of clinic days by modeling the rate of cases per clinic day.

Counts of TVT and skin treatment cases per program were used as outcome variables. Calendar year was the primary predictor and was treated as a continuous variable to estimate the annual multiplicative change in rate. Negative binomial models were selected due to overdispersion in the count data; the dispersion parameters were 4.69 for TVT and 10.55 for skin treatments.

An initial attempt to include program as a random intercept using GLMM was abandoned because between-program variance was estimated to be zero, indicating no additional heterogeneity after adjustment for sampling days. Therefore, fixed-effects model results are reported. Model fit was assessed using deviance residuals and dispersion statistics.

For visualization and interpretation, annual rates were standardized to cases per 7 clinic days, calculated as sum(cases)/sum(days) × 7, to represent a typical program week and reduce stochastic variation associated with single-day rates. All analyses were performed in R v4.3.0 using the MASS package. Statistical significance was set at α = 0.05.

**Figure 8:**
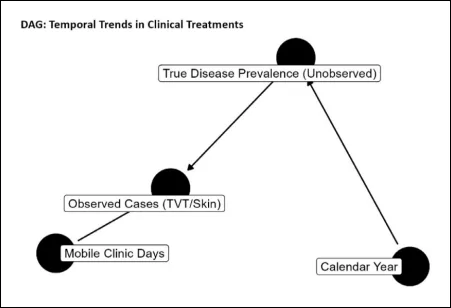
Directed acyclic graph showing temporal trends in transmissible venereal tumor (TVT) and skin treatments.

A DAG mapping the relationships among calendar year, sampling effort, and observed cases was formulated to justify the approach used for modeling temporal trends in TVT and skin treatments. The DAG (Figure 8) identifies Calendar Year as the primary exposure driving unobserved true disease prevalence. Because mobile clinic days may directly constrain observed counts but do not causally alter the true underlying prevalence, they were modeled as an offset, log(days), rather than as a covariate. Although unmeasured program-level factors were initially hypothesized to confound observed counts, preliminary GLMM analysis indicated zero between-program variance. Consequently, the DAG reflects a final causal structure without program-level latent confounders, justifying the use of fixed-effects negative binomial regression to estimate the annual multiplicative change in treatment rates.

**Approximate benefit-cost analysis:** Operational costs were calculated based on the operational expenditure of recent programs, including labor, transport, surgical supplies and drugs, food, and accommodation, while excluding service charges. The cost of post-exposure prophylaxis and the total cost of rabies were derived from cited sources, and the current dog population of Sri Lanka was estimated as one-eighth of the estimated human population.

## RESULTS

**Changes in the sexually active dog population of the Anuradhapura Municipality from 2021 to 2025**

Table 3 summarizes the results of the negative binomial regression analyses for nine demographic categories: total dog count; males; females; owned dogs; community dogs (free-roaming dogs cared for collectively by the community); owned males; owned females; community males; and community females.

**Table 3:** Effort-adjusted annual trends in catch per day, 2021–2025*.

**Response**	**IRR_per_year**	**95% ** **CI**	**p-value**	**Percentage changes**	**Theta (Standard error)**	**Resid_dev_df**
Total	1.03	0.93–1.14	0.55488235	3.0	42.05 (27.18)	1.67
Male	0.903	0.816–0.998	0.03622964*	-9.7	46.42 (32.47)	1.67
Female	1.08	0.97–1.21	0.14899790	8.1	35.59 (23.07)	1.68
Owned	1.15	0.98–1.35	0.07018171	14.7	17.91 (11.54)	1.68
Owned Male	1.03	0.81–1.30	0.79099748	2.8	9.76 (6.57)	1.68
Owned Female	1.24	1.03–1.49	0.01069109*	24.1	14.33 (9.20)	1.69
Community Dog	0.963	0.88–1.06	0.41294902	-3.7	50.74 (33.51)	1.66
Community Male	0.953	0.81–1.12	0.53483703	-4.7	17.76 (11.68)	1.66
Community Female	0.969	0.90–1.04	0.35403199	-3.1	96.65 (69.54)	1.67

(IRR = incidence rate ratio per calendar year from negative binomial regression with log(catching_days) offset. CI = Confidence interval. Theta = dispersion parameter. Residual dev/df ∼1 indicates adequate fit. DHARMa tests showed no overdispersion or zero-inflation for any model (all p > 0.3). The asterisk (*) indicates significant p- values.)

**After adjustment for the number of catching days, total dog counts (IRR = 1.03, 95% CI:** 0.93–1.14, p = 0.55), owned dogs (IRR = 1.15, 95% CI: 0.98–1.35, p = 0.07), and females (IRR = 1.08, 95% CI: 0.97–1.21, p = 0.15) showed no significant temporal changes (Table 3). Although the point estimates indicated annual increases of 3.0% for total dogs and 8.1% for females, these trends were not statistically significant (p > 0.05).

**In contrast, the number of male dogs declined significantly over time (IRR = 0.903, 95% CI:** 0.816–0.998, p = 0.036), corresponding to an annual reduction of 9.7%.

Despite slight decreases in point estimates, no significant temporal trends were observed for community dogs (IRR = 0.963, 95% CI: 0.88–1.06, p = 0.413), community males (IRR = 0.953, 95% CI: 0.81–1.12, p = 0.535), or community females (IRR = 0.969, 95% CI: 0.90–1.04, p = 0.354).

Among the owned population, owned females exhibited a significant increasing trend, with an annual increase of 24.1% (IRR = 1.24, 95% CI: 1.03–1.49, p = 0.011). No significant trend was observed for owned males (IRR = 1.03, 95% CI: 0.81–1.30, p = 0.791).

Figures 9A–9I illustrate annual counts and effort-adjusted trends for the nine demographic groups from 2021 to 2025.

**Figure 9A:**
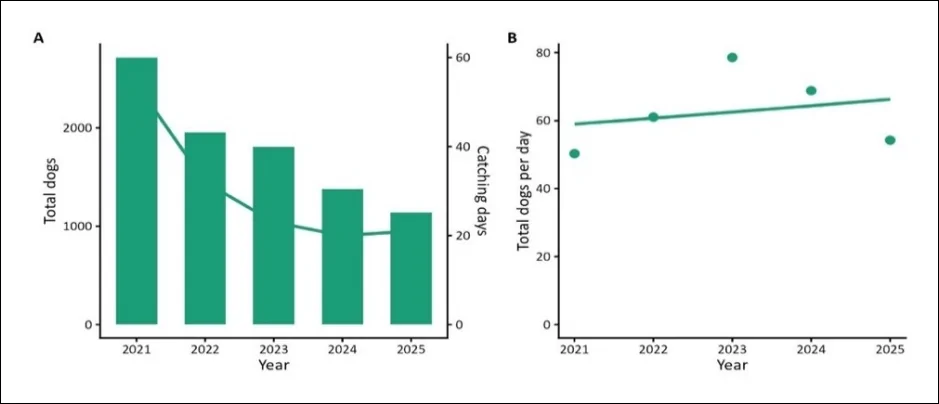
Annual captures and effort-adjusted trends for the total dog population, 2021–2025.

**Figure 9B:**
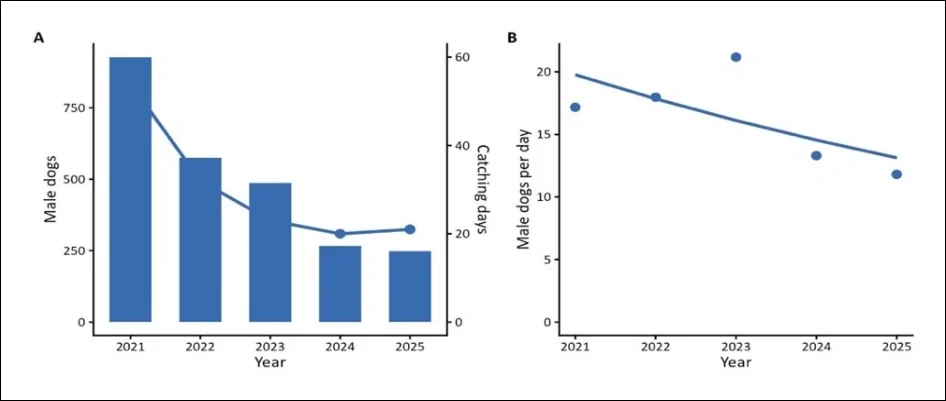
Annual captures and effort-adjusted trends for the total male dog population, 2021–2025.

**Figure 9C:**
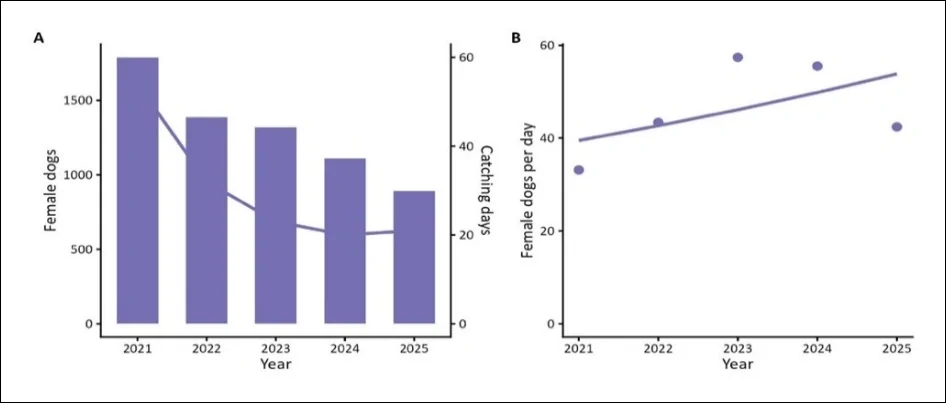
Annual captures and effort-adjusted trends for the total female dog population, 2021–2025.

**Figure 9D:**
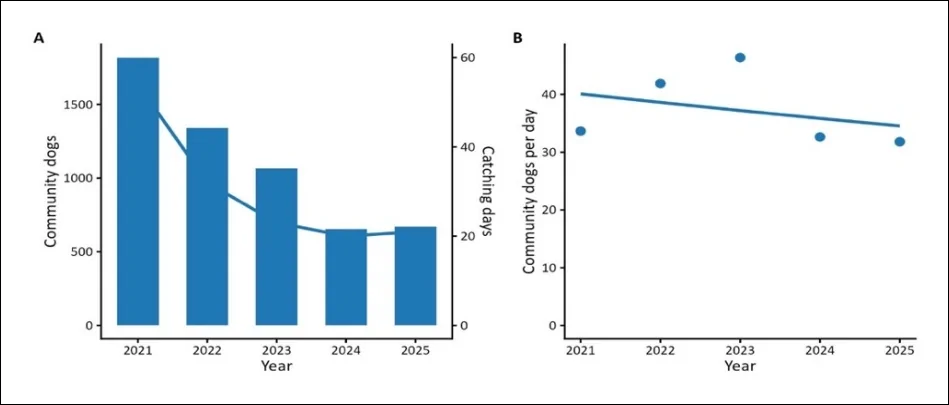
Annual captures and effort-adjusted trends for the community dog population, 2021–2025.

**Figure 9E:**
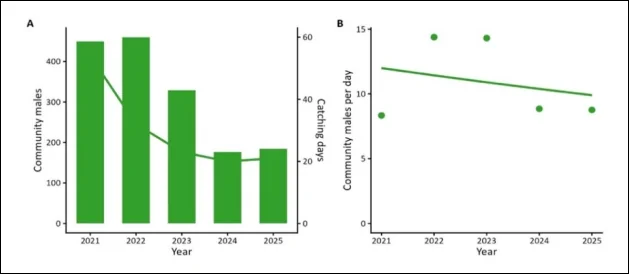
Annual captures and effort-adjusted trends for the male community dog population, 2021–2025.

**Figure 9F:**
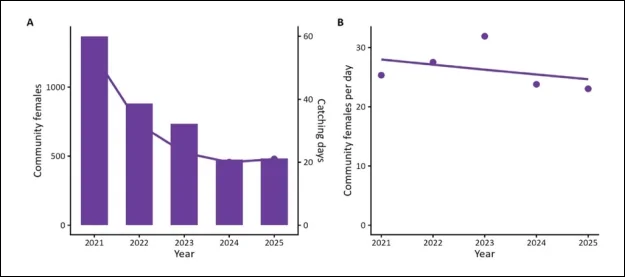
Annual captures and effort-adjusted trends for the female community dog population, 2021–2025.

**Figure 9G:**
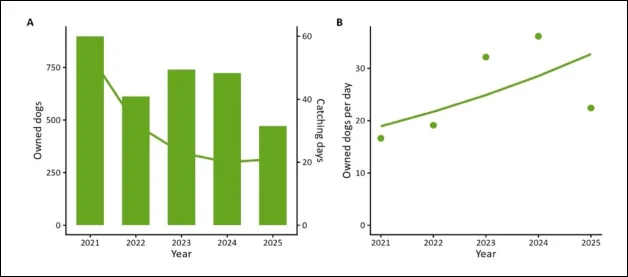
Annual captures and effort-adjusted trends for the owned dog population, 2021–2025.

**Figure 9H:**
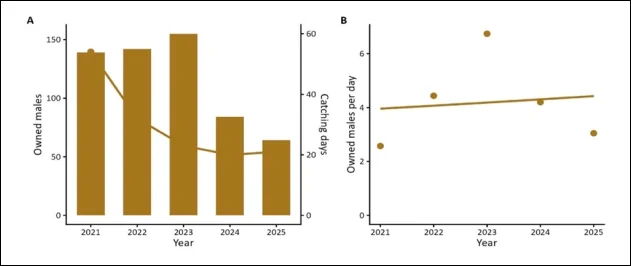
Annual captures and effort-adjusted trends for the owned male dog population, 2021–2025.

**Figure 9I:**
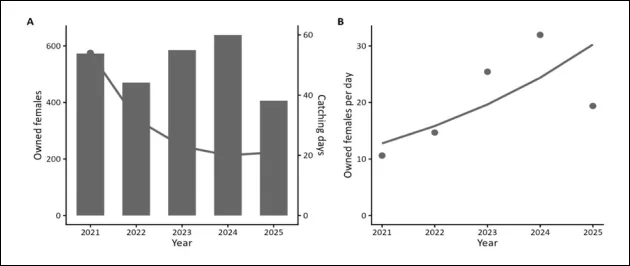
Annual captures and effort-adjusted trends for the owned female dog population, 2021–2025.

For Figures 9A–9I, the left axis shows annual counts as bar charts, whereas the right axis shows the number of catching days as a linear trend. Points represent observed rates, and lines represent fitted values from negative binomial regression models with log(number of days) included as an offset. Colors were kept consistent within categories. Although raw counts appeared to decrease across categories, significant effort-adjusted annual trends were detected only for male dogs and owned females (Table 3).

**Changes in sex ratios in the sexually active dog population of the Anuradhapura Municipality from 2021 to 2025**

Linear regression was used to evaluate changes in male:female ratios (Table 4). The overall male:female ratio declined significantly by 0.065 units per year (β = −0.065, p = 0.020), decreasing from 0.518 in 2021 to 0.240 in 2025. In contrast, no significant temporal trends were detected for owned dogs or community dogs (both p > 0.14). Mean ratios indicated a persistent female bias in the total, owned, and community populations throughout the study period.

**Table 4:** Male-female ratios and temporal trends for 2021-2025.

**Category**	**Mean ± Standard deviation**	**Range**	**β per year**	**95% Confidence interval**	**T-test**	**p-value**
Total	0.0364 ± 0.111	0.0240–0.518	–0.065	–0.112 to –0.019	–4.51	0.020*
Owned	0.220 ± 0.072	0.131–0.302	–0.034	–0.091 to 0.022	–1.93	0.149
Community	0.410 ± 0.076	0.329–0.522	–0.005	–0.092 to 0.083	–0.17	0.873

β= slope from ordinary least squares regression of ratio on year. Negative β indicates increasing female bias over time. Ratio <1 indicates a higher proportion of females. The asterisk (*) indicates significant p- values).

**Figure 10:**
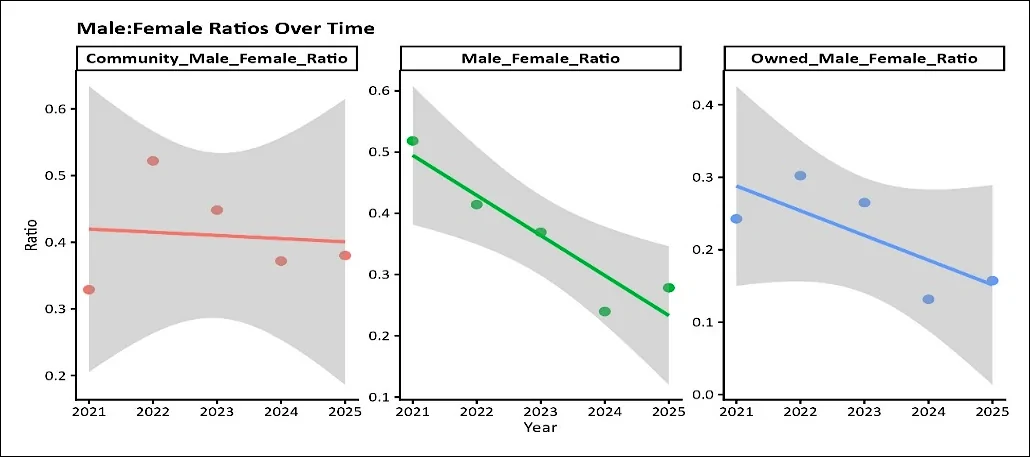
Male:female ratios for total, owned, and community dogs from 2021 to 2025.

Figure 10 illustrates changes in male:female ratios for total, owned, and community dogs.

Figure 10 shows that the overall male:female ratio decreased significantly over time (β = −0.065, 95% CI: −0.112 to −0.019, p = 0.020), indicating an increasing female bias in total captures.

**No evidence of linear temporal trends was detected for owned dogs (β = −0.034, 95% CI:** −0.090 to 0.022, p = 0.149) or community dogs (β = −0.005, 95% CI: −0.092 to 0.083, p = 0.873).

The mean male:female ratios over the study period were 0.364 ± 0.111 for total dogs, 0.220 ± 0.072 for owned dogs, and 0.410 ± 0.076 for community dogs. All ratios remained below 1.0 throughout the study period, indicating a persistent female bias in the overall population, as well as in owned and community dogs. The significant decline in the total ratio reflected a shift from approximately 1 male per 2.1 females in 2021 to 1 male per 3.6 females in 2025.

All regression models satisfied the assumptions of linear regression, and no evidence of residual autocorrelation was detected.

### Population trends for different age groups

Age groups of owned and free-roaming dogs presented for vaccination and sterilization were determined primarily by dental aging, supplemented by other visual characteristics. Dogs were categorized into four age groups: <3 months, 3 months–1 year, 1–6 years, and >6 years.

Age-specific capture rates showed no significant annual trends after adjustment for the number of catching days (Table 5; all p > 0.18). Nevertheless, point estimates suggested annual declines in puppies <3 months (IRR = 0.753, corresponding to a 24.7% decrease per year) and senior dogs >6 years (IRR = 0.846, corresponding to a 15.4% decrease per year), whereas older puppies aged 3 months–1 year showed an estimated annual increase of 33.1% (IRR = 1.33). Adults of reproductive age (1–6 years) remained relatively stable, with an estimated annual decline of 6.24% (IRR = 0.938). All models demonstrated adequate fit and showed no evidence of overdispersion or zero-inflation.

**Table 5:** Annual trends in age-specific counts per day, 2021–2025.

**Age group**	**IRR per year**	**95% Confidence interval**	**% Change per year**	**p-value**	**Resid dev/** **df**
<3 months	0.753	0.467–1.21	–24.7	0.311	2.21
3 months- 1 year	1.33	0.905–1.94	+33.1	0.185	1.80
1 year- 6 months	0.938	0.622–1.39	–6.24	0.752	1.78
>6 years	0.846	0.597–1.19	–15.4	0.358	1.73

IRR = incidence rate ratio from negative binomial generalized linear model with log (number of days) offset. All models n = 5 years. No evidence of overdispersion or zero-inflation (all DHARMa p > 0.4).

**Figure 11:**
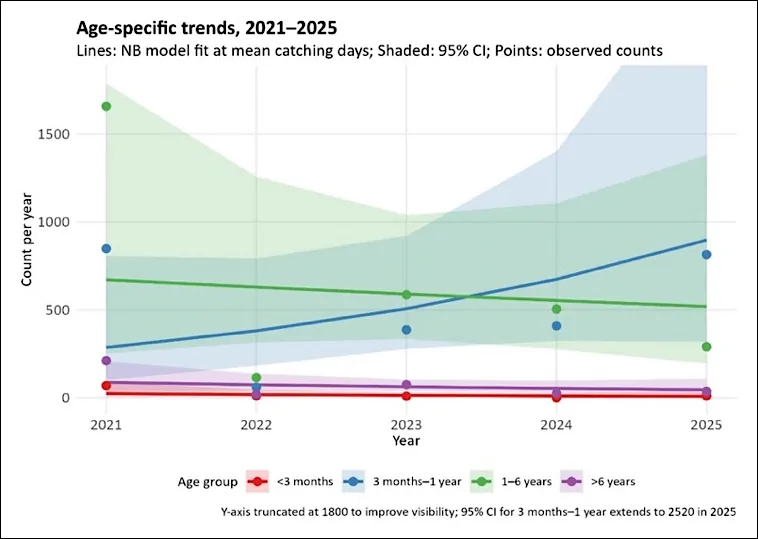
Age-specific trends of dogs in Anuradhapura, Sri Lanka, from 2021 to 2025.

Observed counts decreased sharply across all age groups in 2022, coinciding with a reduction in the number of catching days (Figure 11). After adjustment for effort, negative binomial models showed no significant temporal trends for any age group (Table 5). Predicted trends evaluated at the mean number of catching days indicated a non-significant increase of 33.1% among older puppies aged 3 months–1 year and declines of 24.7% and 15.4% among puppies <3 months and dogs >6 years, respectively. Adults of reproductive age (1–6 years) showed relatively little change. The wide CIs reflected the uncertainty associated with the limited number of annual observations (n = 5).

### Influence of the buffer zone on reducing the population within the municipality

Buffer zones have been widely used in wildlife conservation and disease monitoring to create geographical barriers around vulnerable populations. In this study, the buffer zone refers specifically to the area outside the municipal boundary that was intensively covered to prevent the influx of unvaccinated and sexually intact dogs into the city through migration and deliberate dumping.

Figure 12A shows the zone-wise population patterns from 2021 to 2025. Counts appeared to stabilize in Zones 1 and 2 before intensified buffer zone coverage, whereas Zone 3 appeared to stabilize during 2024 and 2025, coinciding with increased buffer zone coverage.

**Figure 12A:**
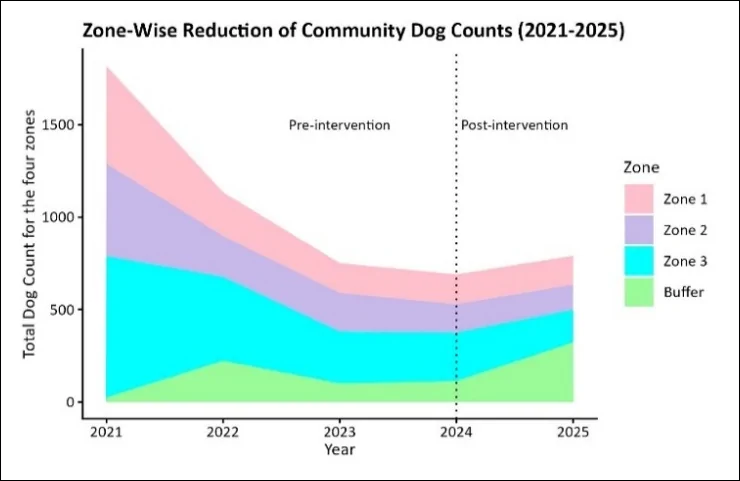
Observed zone-wise trends for the community dog population from 2021 to 2025.

However, total catch rates did not change significantly after 2023 in any zone (Table 6A). Zone 1 declined by 8.4% (ratio = 0.916, p = 0.461), Zone 2 increased by 6.1% (ratio = 1.061, p = 0.611), and Zone 3 declined by 4.1% (ratio = 0.959, p = 0.685), but all p-values exceeded 0.05.

**Table 6A:** Zone-wise change in total catch rate after 2023.

**Zone**	**Pre rate**	**Post rate**	**Ratio**	**% Change**	**p-value**	**95% Confidence interval**
1	33.5	30.7	0.916	-8.4%	0.461	0.724-1.16
2	28.9	30.7	1.061	+6.1%	0.611	0.844-1.33
3	33.8	32.4	0.959	-4.1%	0.685	0.782-1.17

Rate = dogs per day. Ratio = Post/Pre from Negative Binomial Generalized linear model.

Similarly, sex-stratified before/after analyses showed no significant post-2023 changes (Table 6B). Male counts remained stable across zones (all p > 0.67), as did female counts (all p > 0.53).

**Table 6B:** Zone-wise change in catch rate after 2023 by sex.

**Zone**	**Sex**	**Pre rate**	**Post rate**	**Ratio**	**% Change**	**p-value**	**95% Confidence interval**
1	Male	9.95	10.00	0.964	–3.6%	0.835	0.682–1.36
2	Male	10.20	10.80	1.080	+7.6%	0.676	0.762–1.52
3	Male	8.75	8.59	0.978	–2.2%	0.890	0.715–1.34
1	Female	23.50	26.80	1.100	+10.0%	0.530	0.817–1.48
2	Female	28.30	26.60	0.924	–7.6%	0.601	0.685–1.25
3	Female	22.80	21.20	0.913	–8.7%	0.531	0.686–1.22

However, buffer spillover effects were sex- and zone-specific (Table 6C). Each additional dog sterilized from the buffer reduced Zone 1 male catch by 0.45% (IRR = 0.9955, 95% CI: 0.9913–0.9995, p = 0.038) and Zone 3 female catch by 0.57% (IRR = 0.9943, 95% CI: 0.9912–0.9975, p = 0.0003). No significant spillover effects were detected for Zone 2, Zone 1 females, or Zone 3 males.

**Table 6C:** Buffer spillover effect on zone catch by sex after 2023.

**Sex**	**Zone**	**Coefficient**	**Incidence rate ratio** ** per buffer dog**	**p-value**	**95% Confidence interval**
Male	1	–0.00455	0.9955	0.038*	0.9913–0.9995
Male	2	–0.00241	0.9976	0.273	0.9932–1.002
Male	3	–0.00217	0.9978	0.224	0.9943–1.001
Female	1	–0.00253	0.9975	0.124	0.9945–1.001
Female	2	–0.00168	0.9983	0.327	0.9950–1.002
Female	3	–0.00568	0.9943	0.0003***	0.9912–0.9975

**Figure 12B:**
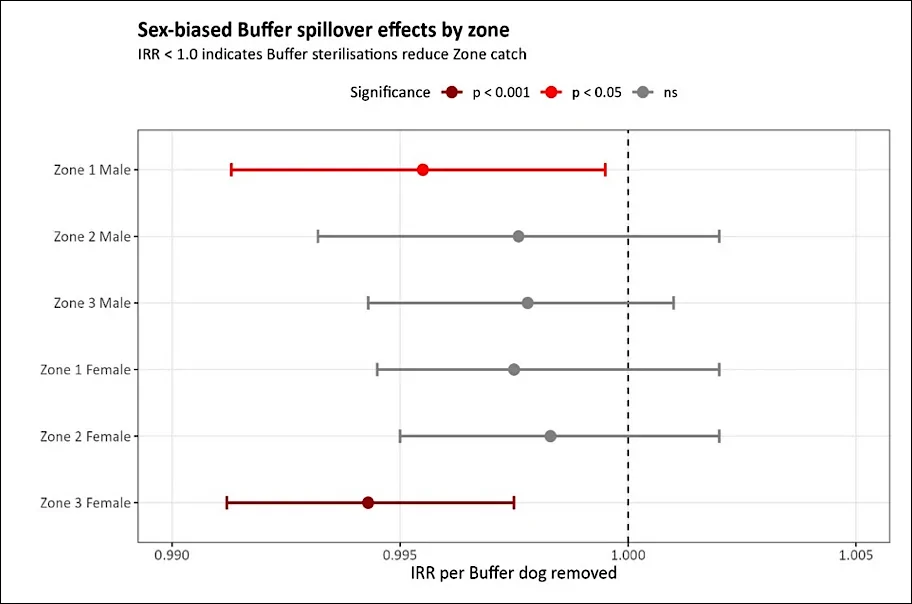
Sex-biased buffer spillover effects by zone.

When scaled to annual sterilization totals, buffer effects were biologically meaningful for the two significant groups (Figure 12C). At the mean annual buffer sterilization rate of 60 dogs, predicted reductions were 23.7% for Zone 1 males (95% CI: 2.9%–40.8%) and 29.2% for Zone 3 females (95% CI: 14.0%–41.1%). At 100 buffer dogs sterilized per year, predicted reductions increased to 36.3% for Zone 1 males (95% CI: 4.9%–59.1%) and 43.4% for Zone 3 females (95% CI: 22.1%–60.5%).

No evidence was found for a delayed cascade effect. Covering the buffer zone during the previous program did not predict the total count in Zone 1 (IRR = 1.38, p = 0.102), and the gap length between buffer and Zone 1 programs was also not significant (p = 0.234). Results were non-significant for both males (buffer presence p = 0.112) and females (buffer presence p = 0.186). Model Akaike information criterion values were higher for lag models (419.8) than for contemporaneous models (398.3), indicating no evidence of delayed displacement.

### Percentage of owned dogs vaccinated and sterilized by October 2025

A questionnaire survey conducted at vaccination and sterilization centers within and outside the municipality from late September to mid-October 2025 was used to estimate vaccination and sterilization coverage among owned dogs. The survey collected 1123 responses from 1687 pet owners who visited the clinics, including 737 from 41 locations within the municipality and 386 from 8 locations outside the municipal boundary. The response rate was 66.57%. Of the total respondents, 912 (approximately 81%) owned at least one dog, representing 1679 owned dogs. Table 7 summarizes vaccination and sterilization coverage achieved by the end of 2025 within and outside the municipality.

**Figure 12C:**
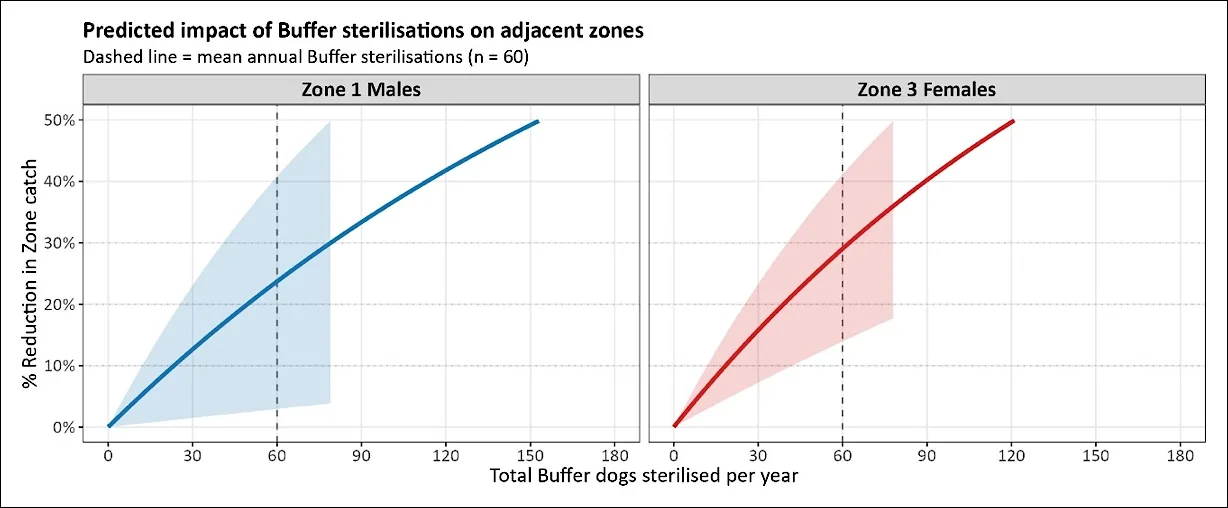
Predicted impact of buffer sterilizations on adjacent zones.

**Table 7:** Sterilization and anti-rabies vaccination coverage among owned dog populations living within and outside the municipality.

**Coverage as ** **a ** **percentage**	**Within the municipality**	**Outside the municipality**
Percentage of sterilized dogs	55.17928287	50.96296296
Sterilized dogs as a percentage of adult animals*	60.41439477	61.42857143
Sterilized female dogs percentage of all females	73.11608961	73.86759582
Sterilized female dogs as a percentage of adult females	78.04347826	86.53061224
Sterilized male dogs as a percentage of all males	37.64478764	30.31914894
Sterilized male dogs as a percentage of adult males	42.66958425	41.9047619
Percentage of dogs vaccinated at least once	94.22310757	83.11111111
Percentage of dogs vaccinated annually	74.20318725	68.88888889
Dog population density (dogs per dog-owning household)	1.701694915	2.096273292

*Adults refer to dogs >5 months, mature enough to be sterilized

By October 2025, 74.2% of owned dogs within the municipality had received annual rabies vaccination for at least three consecutive years. Owned dogs being vaccinated for the first time and those vaccinated irregularly across years were recorded as separate categories. Annual vaccination coverage outside the municipal boundary was 68.9%, close to the 70% target set by the World Health Organization for establishing herd immunity against rabies [35]. When dogs vaccinated against rabies at least once were considered, vaccination coverage increased to 94.2% within the municipality and 83.1% outside the municipal boundary.

The percentage of sterilized adult owned dogs was 60.4% within the municipality and 61.4% outside the municipality. Sterilization coverage among adult females was 78.0% within the municipal boundary and 86.5% outside it. Adult male sterilization coverage was approximately 42% both within and outside the municipality.

### Influence of the number of dogs per household on regular ARV and sterilization

Crude sterilization coverage ranged from 0.0% to 100.0% by location. Negative binomial regression indicated that centers with higher mean numbers of dogs per household significantly vaccinated and sterilized fewer dogs overall (Table 8A).

**Table 8A:** Negative binomial regression of vaccinated and sterilized dog counts at 49 centers.

**Outcome**	**Predictor**	**β**	**Standard error**	**95% ** **CI**	**IRR**	**95% CI IRR**	**p-value**
Vaccinated dogs	No. of dogs per household	–0.237	0.069	–0.372 to –0.102	0.789	0.689–0.903	<0.001*
Sterilized dogs	No. of dogs per household	–0.306	0.149	–0.598 to –0.014	0.786	0.550–0.986	0.040*

*Indicates significant p-values; p ≤ 0.05, CI = Confidence interval, IRR = Incidence rate ratio.

Each additional dog per household predicted a 21.1% reduction in total vaccinated dogs (IRR = 0.789, 95% CI: 0.689–0.903, p < 0.001). A similar pattern was observed for sterilization, where each additional dog per household was associated with a 21.4% reduction in the probability of sterilization (IRR = 0.786, 95% CI: 0.550–0.986, p = 0.040), as shown in Figure 13A.

**Figure 13A:**
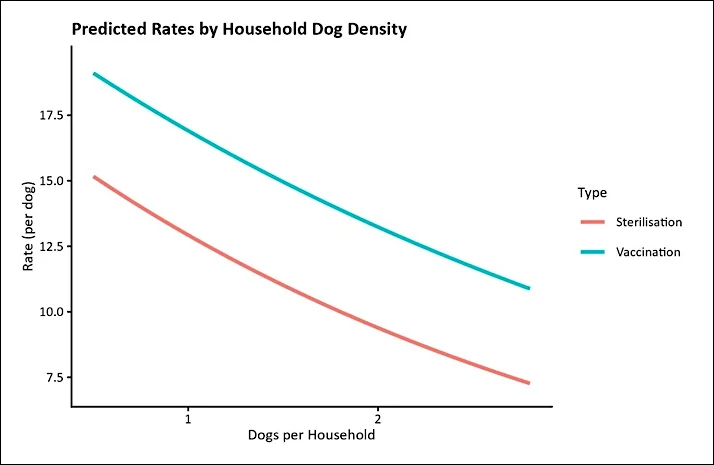
Association between mean dogs per household and location-level vaccination and sterilization rates.

The aggregate result suggested that locations with larger dog-owning households had poorer outcomes, supporting the hypothesis that higher numbers of dogs per household may reduce annual vaccination and sterilization uptake. However, GLMM using dog-level data from 865 of the 912 dog-owning households, after excluding rows with mismatched data, showed a contrasting pattern. Between-location variance was substantial for both outcomes. For vaccination, MOR = 1.69 and ICC = 0.07, indicating that the median odds of vaccination differed by 69% between high- and low-coverage locations for households with similar numbers of pets. Heterogeneity was greater for sterilization, with MOR = 2.43 and ICC = 0.17, indicating a 2.4-fold median difference in odds between locations. This between-location variance exceeded the household-level effect of dog number, indicating that coverage differences were driven more by location context than by household size.

**Figure 13B:**
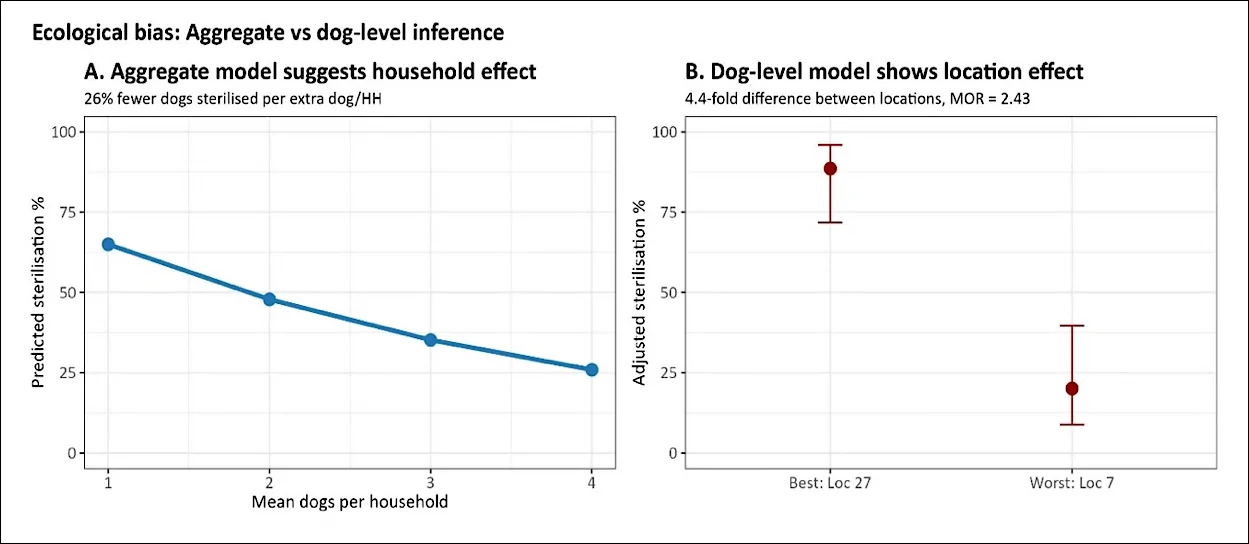
Aggregate-level versus household-level inference.

While the aggregate model-predicted an approximate one-quarter reduction for each additional dog per household, individual-level responses showed strong variation among locations, including an almost four-fold gap between the locations with the best and worst sterilization performance.

**Adjusted sterilization probabilities ranged from 20.1% (95% CI:** 8.8%–39.7%) in Location 7 to 88.6% (95% CI: 71.8%–96.0%) in Location 27. Supplementary Table 1 provides vaccination and sterilization percentages and ranks for each location.

Sterilization showed a wider spread and larger CIs than vaccination, consistent with the higher MOR. Several locations with small sample sizes, such as Location 4 (n = 2 households), had wide CIs spanning 19.6%–83.7%, indicating that their ranks were unreliable despite extreme point estimates. The graphical patterns showed greater between-location heterogeneity for sterilization than for vaccination, as indicated by the broader distribution of points, and a lower mean rate for sterilization than for vaccination, as indicated by the dotted reference line.

**Six locations met the priority criteria:** adjusted sterilization coverage <35% and an upper 95% CI <50% (Table 8B).

### Reported cases of TVT and skin treatments in Anuradhapura Municipality from 2022 to 2025

A total of 286 TVT cases and 2409 skin treatment cases were recorded in the Anuradhapura Municipality across 14 programs between 2022 and 2025, covering 83 mobile clinic days. Table 9A summarizes the annual counts, sampling days, and observed rates of TVT and skin treatment cases from 2022 to 2025. Data for the base year 2021 were unavailable.

**Figure 13C:**
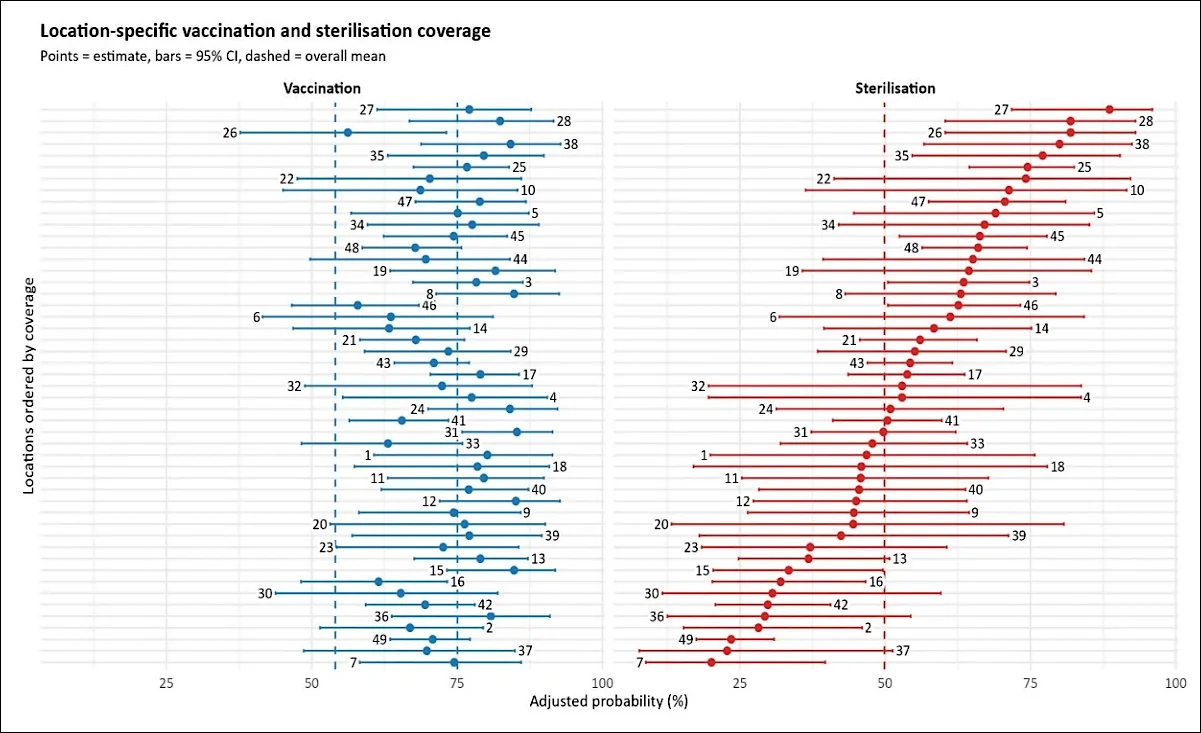
Caterpillar plots of adjusted vaccination and sterilization probabilities by location.

**Table 8B:** High-priority locations with the lowest sterilization coverage.

**Location no.**	**No. of households**	**Adjusted sterilization Confidence interval**
2	12	28.2% (15.3–46)
7	8	20.1% (8.8–39.7)
15	24	33.4% (20.4–49.6)
16	27	32% (20.2–46.6)
42	43	29.8% (20.8–40.6)
49	52	23.5% (17.5–30.9)

**Figure 13D:**
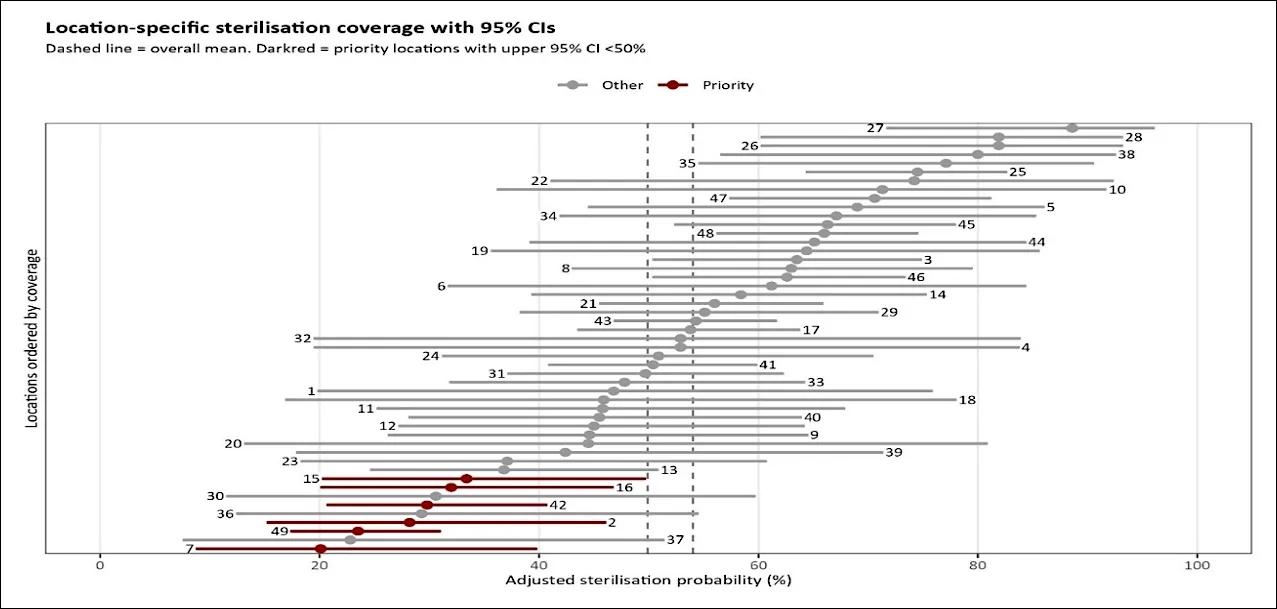
High-priority locations for sterilization based on adjusted sterilization probability.

**Table 9A:** Annual counts, sampling days, and observed rates of transmissible venereal tumor (TVT) and skin treatments, 2022–2025.

**Year**	**Program**	**Municipal council days**	**TVT cases**	**Skin cases**	**TVT/7 days**	**Skin cases/ 7 days**
2022	5	25	77	571	21.6	159.9
2023	3	20	73	635	25.6	222.3
2024	3	18	69	585	26.8	227.5
2025	3	16	67	618	29.3	270.4

After adjustment for the number of clinic days per program, TVT cases increased by 9.8% per year; however, this trend was not statistically significant (IRR = 1.098, 95% CI: 0.893–1.355, p = 0.373). In contrast, skin treatments increased significantly by 18.8% per year (IRR = 1.188, 95% CI: 1.067–1.324, p = 0.001). Table 9B summarizes the negative binomial regression results.

**Table 9B:** Negative binomial regression results for annual trends in transmissible venereal tumor (TVT) and skin treatment rates, 2022–2025.

**Outcome**	**Incidence rate ratio** ** per year**	**% Change per year**	**95% Confidence interval**	**p-value**	**Dispersion**
TVT	1.098	9.8	0.893–1.355	0.373	4.69
Skin treatment	1.188	18.8	1.067–1.324	0.001*	10.55

**Figure 14:**
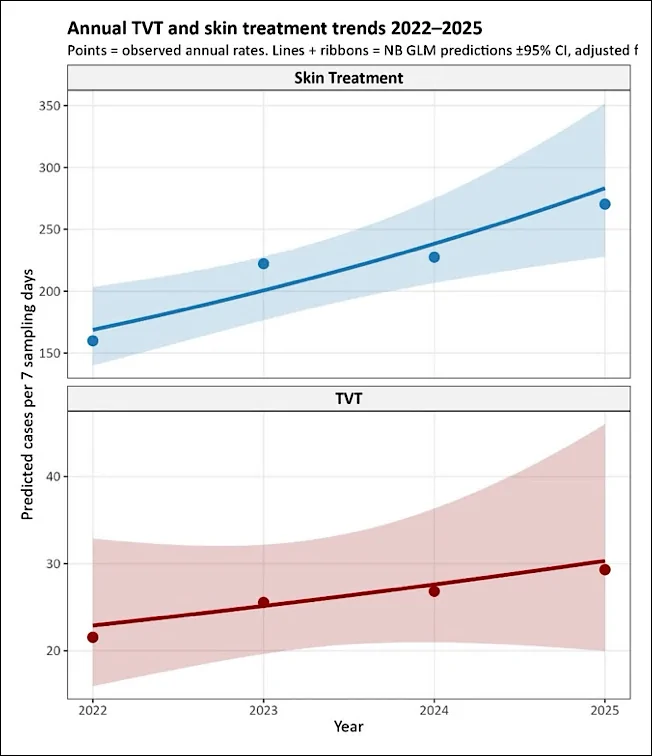
Annual rates of canine TVT and skin treatments per 7 mobile clinic days, 2022–2025. Points represent observed annual rates calculated as total cases divided by total clinic days × 7. Lines and shaded areas show predicted trends and 95% confidence intervals from negative binomial regression models with log(clinic days) as an offset. Skin treatments increased significantly by 18.8% per year (p = 0.001), whereas TVT increased by 9.8% per year, although the trend was not significant (p = 0.373).

Figure 14 shows observed annual rates per 7 clinic days with model-predicted trends and 95% CIs. The predicted TVT rate remained relatively stable across years, whereas the skin treatment rate showed a pronounced upward trajectory.

### Other indicators of welfare

Deaths due to postoperative complications remained low, with only 60 canine deaths reported during the entire program, all related to aspiration. This accounted for <1% of all surgeries performed, indicating a high level of perioperative welfare. Postoperative edema following surgical castration was reported to the Municipal Veterinary Office for a limited number of male dogs; however, the exact number was unavailable to the authors.

### Regional incidence of dog bites, human ARV, and rabies-related human deaths

The national health data system does not maintain records of dog bites, human ARV, or human rabies deaths at the local government level, preventing a comprehensive assessment of the program’s contribution to reducing rabies incidence. However, according to unpublished Ministry of Health data, seven rabies-related human deaths were reported from Anuradhapura District between 2020 and 2023: three in 2020, one in 2021, one in 2022, and two in 2023. No rabies-related human deaths were reported from the district in 2024 or 2025. District-specific statistics for human post-exposure prophylaxis could not be obtained.

### Analysis of monetary benefits and costs

Each program treated an average of 35 dogs at a daily operational cost of 52,600 LKR (164 USD). This included drugs and surgical supplies (2000 LKR), daily wages for six assistants (1000 LKR each), meals and accommodation for six assistants and two veterinarians (three meals per day at 400 LKR each and 15,000 LKR for accommodation), and transport costs (20,000 LKR). The calculation excluded veterinary service charges because government veterinarians conducting similar programs would likely receive fixed monthly salaries rather than daily or project-based payments. Therefore, the average per dog cost for vaccination and sterilization was approximately 1500 LKR (4.68 USD). The actual per dog cost may be slightly lower because one to ten cat surgeries were also performed each day.

With the Sri Lankan human population currently estimated at 23.35 million, the national dog population, based on a 1:8 dog-to-human ratio, would be approximately 2.92 million. Considering an annual expenditure of 700 million LKR on human post-exposure prophylaxis alone, the per dog burden of human post-exposure prophylaxis, under a hypothetical scenario in which no dogs were vaccinated or sterilized and dogs were responsible for all bites, would be approximately 240 LKR (0.75 USD).

The Global Alliance for Rabies Control estimates the annual cost of rabies in Sri Lanka at 21,122,199 USD (6,759,103,680 LKR). Accordingly, the per dog cost of rabies would be approximately 2315 LKR (7.23 USD), which exceeds the per dog cost of the intervention. Based on these estimates, the benefit-cost ratio of the intervention is approximately 1.5.

## DISCUSSION

### Novel aspects of the study

The novelty of this study lies in the fact that it represents the longest (>5 years) CNVR study conducted in a culturally challenging religious city in Asia and one of the few studies that simultaneously evaluated owned and free-roaming populations, including community, stray, and feral dogs, as well as both sexes. Furthermore, this study introduces and validates the concept of using a buffer zone to reduce dumping pressure, which is novel in the context of domestic dog population management despite being widely adopted in epidemiology and wildlife management, including wild dog populations. The study also investigated the relationship between the number of dogs per household in low-income communities and the likelihood of sterilization and regular ARV.

**Shifts in counts and sex ratios of owned and community dogs from 2021 to 2025 and the increasing** **willingness of low-income communities to adopt female dogs**

Using capture effort and clinical intake as proxies provides only limited insights into the actual dog population within the Anuradhapura Municipality. However, based on the nationally accepted dog:human ratio of 1:8 and the current human population of 63,276, the estimated dog population is approximately 7910, suggesting that the observations reasonably represent the local context.

Table 10 summarizes the annual numbers of sterilized dogs from 2020 to 2025 as percentages of the estimated population.

**Table 10:** Counts and percentages of sterilized dogs in Anuradhapura Municipality from 2020 to 2025.

**Year**	**Sterilized dog count**	**Percentage**
2020	669	8.46%
2021	2716	34.34%
2022	1953	24.69%
2023	1085	13.72%
2024	1376	17.39%
2025	1139	14.40%

The cumulative number of sterilized dogs over the 6-year period, which included only sexually mature adults, reached 10,334, exceeding the estimated population by 2424 animals (30.64%). When younger age groups are considered, the true population is likely to exceed this estimate. Given the high dog density observed before and during the initial stages of the project, it is probable that the actual dog:human ratio in Anuradhapura was considerably higher than the national estimate. This discrepancy highlights the need for region-specific population estimates when planning dog population management programs.

Although total dog counts declined over time, except for a sudden increase of 21.1% in 2023, no significant temporal trend was observed after adjustment for differences in the number of catching days. It should be emphasized, however, that the reduction in the number of catching days was intentionally implemented by project management in response to the apparent decline in free-roaming dog numbers. Consequently, including the number of days as an offset could partially mask genuine population reductions. Conversely, the absence of a significant increase over a 5-year period itself suggests successful intervention, because the absence of sterilization would normally result in annual population growth. Previous studies have estimated annual increases ranging from 2.5% over 20 years at a 30% female sterilization rate [20] to 8% over 20 years at a 45% female sterilization rate [39]. Although these estimates originate from temperate regions and are highly context dependent, population growth in tropical countries such as Sri Lanka is likely to be higher due to more frequent estrous cycles. The slight declines observed in community dogs (3.7%), community males (4.7%), and community females (3.1%) were not statistically significant. In contrast, the marginal increase observed in owned dogs (14.7%) suggests improving responsible pet ownership. Interestingly, female ownership increased significantly by 24.1%, whereas the total male population declined by 9.7%.

These sex-specific changes were also reflected in the changing sex ratios. The overall male:female ratio declined significantly, whereas no significant changes were observed in either the owned or community categories. This apparent contradiction may represent Simpson's paradox, suggesting that the overall shift was generalized rather than driven by a specific subgroup. Because many owned male dogs roam freely without collars, some males classified as free-roaming during capture may actually have had owners.

Although the exact causes of the decline in male numbers remain unclear, a decreasing preference for adopting male puppies may have contributed. Male migration patterns, discussed later in relation to the buffer zone, may also have influenced male abundance within the municipality. In contrast, the significant 24.1% increase in the number of owned females strongly suggests increased female adoption. Historically, female puppies were more likely to be abandoned and less likely to be adopted, resulting in higher mortality caused by poor welfare and road accidents. Since the cost of sterilization currently exceeds 5000 LKR (~16 USD), many low-income households cannot afford these procedures. Because owners of female dogs bear the financial burden associated with unwanted litters, male dogs have traditionally been preferred. In addition, perceptions that intact males are better guard dogs and cultural beliefs among Sri Lankan Buddhists that sterilization is sinful and may affect fertility in the next life may contribute to resistance against male sterilization.

This preference for males remains evident from the survey findings. Within the municipality, there were 518 owned males and 491 owned females, whereas outside the municipality, there were 376 owned males and 287 owned females. Furthermore, only approximately 42% of adult males were sterilized in both regions, whereas the percentage of sterilized adult females exceeded 78%. The first author recalls numerous occasions when owners refused to sterilize male dogs because they wished to preserve natural sexual behavior and were unconcerned about unwanted pregnancies. Nevertheless, the contribution of intact owned males to reproduction among free-roaming females remains unknown.

The demographic changes observed were reflected in the shift in the male:female ratio from 1:2.1 to 1:3.6. Arguably, the 1:0.95 sex ratio among owned dogs within the municipality revealed by the questionnaire survey was more balanced than the 1:0.76 ratio observed outside the municipality, suggesting that improved access to sterilization services enhances the willingness of owners to adopt female dogs. In addition, the Vets for Future team's deliberate efforts to educate the public about the advantages of female dogs, including their perceived loyalty to families, may have contributed to this shift.

Previous dog population management studies have rarely examined the effects of interventions on sex ratios, and many programs have focused exclusively on females [28, 40]. Furthermore, the female-biased free-roaming population observed in this study contrasts with findings from other South Asian regions reporting approximately equal sex ratios [31, 41]. However, a preference for male pets has been documented in Bhutan, where the reported sex ratio of 1.4:1 is comparable to the present study's findings.

### Impact of the CNVR effort on different age groups

Age-specific analyses did not reveal statistically significant trends at α = 0.05. Nevertheless, puppies aged <3 months exhibited an estimated annual decline of 24.7%, whereas the reduction among sexually active adults aged 1–6 years was only 6.24%. The 15.4% decline among dogs >6 years old is likely attributable to natural mortality, because this age group was not specifically targeted by the intervention.

Interestingly, older puppies aged 3 months–1 year showed an estimated 33.1% increase. Because the population captured increasingly consisted of owned dogs and female ownership increased significantly, the expansion of this age group, which is most likely to comprise newly adopted animals, may be viewed positively, as early sterilization prevents unintended pregnancies. Previous modeling studies have suggested that sterilizing females before 1 year of age at rates of approximately 26% may be sufficient to arrest long-term population growth [20]. Although these estimates are context dependent, prioritizing newly adopted young animals may strengthen future interventions.

### Contribution of the buffer zone to controlling the free-roaming dog population within the city

During the first year of the project, the authors recognized that the food resources available to free-roaming dogs were stable due to surplus food from temples, food waste left by pilgrims, and deliberate feeding by volunteers and visitors. Consequently, immigration and deliberate dumping were considered the principal mechanisms responsible for maintaining dog numbers within the city. Therefore, the project boundary was extended by 3 km beyond the municipal boundary in the second year, based on the assumption that this distance approximated the maximum roaming range of free-roaming dogs. Although populations within Zones 1 and 2 stabilized by the third year, the population in Zone 3 did not decline as expected, leading to more intensive buffer zone coverage during 2024 and 2025.

Interestingly, the effects of the buffer zone were sex-specific. Significant effects were detected for Zone 1 males and Zone 3 females, whereas no effects were observed for either sex in Zone 2. The vacuum effect created by sterilization activities within the buffer may have drawn Zone 3 females outward while simultaneously reducing organized dumping of females and unwanted litters into the municipality. The absence of a similar effect among males in Zone 3 may be attributable to the overall decline in males and their lower vulnerability to abandonment.

The lack of detectable effects in Zone 2 and the selective effect on Zone 1 males remain difficult to explain. Nevertheless, several hypotheses may provide a framework for future investigations.

First, although temples within Zone 2 were originally assumed to be the primary dumping sites, truck drivers heading to markets within Zone 1 may preferentially abandon females and female puppies nearby. Once the buffer intervention reduced the supply of sexually mature females, intact males may have migrated outward in search of receptive partners, whereas resident females remained within familiar territories. Notably, the intact dogs remaining in Zone 1 after several years of intervention likely represent particularly timid individuals that repeatedly escaped capture.

Second, because Zone 2 is approximately 1.4 times as large as Zone 1 while maintaining comparable population levels, its lower density and abundant food supply may reduce competition and minimize vacuum effects.

Third, Zone 2 may contain hidden source populations. The Anuradhapura cemetery, located in the southeastern part of Zone 2, is home to a population of highly aggressive feral dogs that are extremely difficult to capture. This large refuge may counteract the buffer zone's influence.

Regardless of the underlying mechanism, these findings support the original hypothesis that peripheral regions immediately beyond target boundaries should be included in dog population management programs to reduce immigration and "seal the boundary." The discovery of sex-specific and zone-specific effects highlights the importance of conducting preliminary studies on dog behavior, migration patterns, and ecological niches before designing intervention zones.

**Figure 15:**
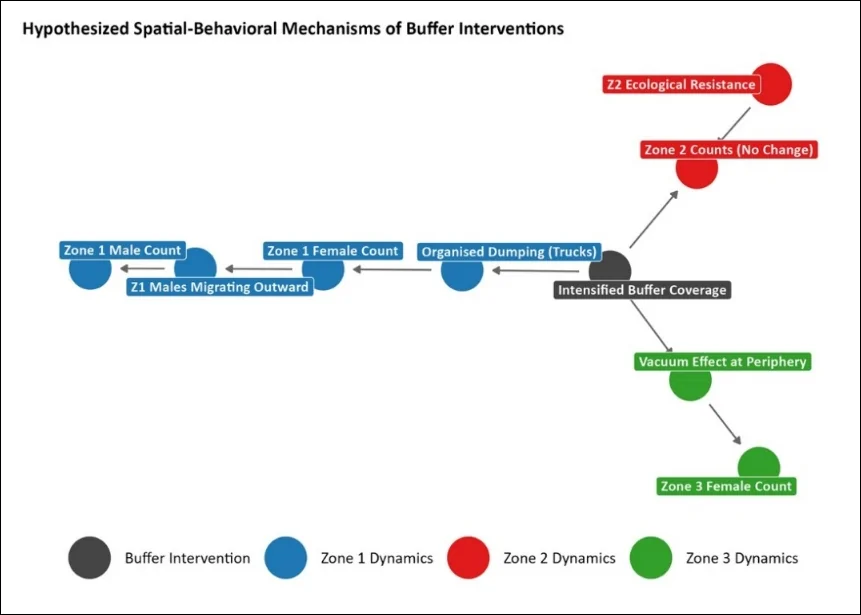
Directed acyclic graph illustrating hypothesized spatial behavioral mechanisms underlying buffer interventions.

A post hoc DAG (Figure 15) was developed to incorporate these unexpected findings and to provide a conceptual framework for future studies. The model integrates the three hypotheses described above and emphasizes the need for microgeographic and niche-based ecological investigations before implementing spatial interventions.

Although buffer zones are widely used in forestry, wildlife management, and disease control programs, this study represents, to the authors' knowledge, the first direct application of this concept to domestic dog population management. Interestingly, the interim Animal Policy of Australia, under Section 10(1)(b) of the Natural Resource Management Act 2004, describes the use of a 35-km external buffer zone around the dog fence within which landowners are expected to eliminate dingoes to protect livestock [36]. Although dingoes and free-roaming domestic dogs belong to the same species (*Canis familiaris*), the distinct behavioral characteristics of Sri Lankan free-roaming dogs make the present application of the buffer zone concept particularly novel.

### ARV and sterilization coverage among owned dogs within and outside the city limits

The survey results revealed that >60% of the adult dogs owned were sterilized. The proportions of sterilized adult females within and outside the municipality were 78% and 86%, respectively, exceeding those reported in comparable interventions conducted elsewhere in South Asia [31]. It should be emphasized that, despite the widespread assumption that sterilizing 70% of a free-roaming population is sufficient to achieve self-sustaining population reduction, the proportion of females requiring annual sterilization depends on the potential population growth rate. This growth rate is determined by adult survival (the proportion of mature females surviving from one breeding season to the next), fecundity (the proportion of mature females producing a litter each breeding season), juvenile survival (the proportion of female pups surviving until sexual maturity), and litter size [35]. These variables are themselves influenced by factors such as food availability, disease outbreaks, and accidents, many of which are unpredictable. Therefore, it is difficult to recommend an ideal sterilization percentage, and continued efforts toward achieving complete sterilization of the free-roaming population may ultimately be required to minimize the suffering of street animals.

Although many dog population management models prioritize female sterilization and largely overlook males, the authors emphasize the importance of sterilizing males for several reasons. First, a sexually mature male dog in Sri Lanka can impregnate multiple females during a breeding season, thereby contributing substantially to population growth. Furthermore, because a considerable proportion of owned and free-roaming females are sterilized, sexually intact males are more likely to experience sexual frustration, which may increase aggression. Such males may extend their roaming range in search of receptive females, resulting in more intense competition among males and increased pressure on the remaining unsterilized females.

Male dogs also contribute substantially to public nuisance through urine marking, territorial fighting, barking, and involvement in motorbike accidents. Sterilization of community males can reduce territorial aggression and nuisance to residents, whereas sterilization of owned males can reduce roaming and fighting behavior, potentially improving their suitability as companion animals.

At present, the authors do not possess sufficient data to estimate the proportion of the free-roaming population that has been sterilized. Nevertheless, zone-specific analyses indicate that the annual number of unsterilized free-roaming dogs requiring surgery has stabilized. Consequently, the authors recommend maintaining CNVR activities twice annually to preserve the gains achieved, given that dogs typically exhibit two reproductive cycles each year. In addition, stronger legal frameworks promoting the sterilization of owned dogs and requiring registration of breeders should be established to ensure that puppies born to owned females do not become unwanted and subsequently abandoned.

Survey data further confirmed that 94% of owned dogs within the municipality and 83% of those outside the municipal boundary had received at least one ARV. The proportions of regularly vaccinated dogs were 74% and 69%, respectively. Since the World Health Organization recommends maintaining vaccination coverage of at least 70%, these findings represent a major achievement in a culturally complex and economically disadvantaged urban environment. The authors therefore propose continuing vaccination campaigns at a frequency similar to that of sterilization programs.

**Influence of the number of dogs per household on the likelihood of regular ARV and sterilization**

Transportation of pets to and from clinics remains a major challenge for low-income households lacking access to personal vehicles. This problem becomes more pronounced in households with multiple dogs, because owners frequently have to leave one or more animals behind. The challenge is particularly severe for sterilization procedures, because postoperative animals require safer and more spacious modes of transportation than routine vaccination visits. These observations led to the initial hypothesis that larger household dog populations would be associated with poorer vaccination and sterilization outcomes. Aggregate-level analyses supported this hypothesis, revealing reductions of 21.4% in sterilization rates and 21.1% in annual vaccination rates for every additional dog per household. This finding challenges the view held by some local health authorities that ARV alone would be sufficient for rabies elimination, as higher dog densities within households were associated with lower vaccination compliance.

However, closer examination of the data revealed that poor outcomes were not always associated with households owning more dogs. Household-level analyses provided another example of Simpson's paradox. Although within-household heterogeneity remained considerable, between-location heterogeneity was highly significant, with adjusted sterilization probabilities ranging from 20.1% to 88.6% in the worst and best-performing locations, respectively. Although the effects of household and neighborhood demographics remain to be explored, factors such as distance to the nearest clinic, availability of transportation, owner education, and attitudes are likely to play important roles. Identifying locations with poor sterilization performance was an important outcome of this analysis and highlights areas requiring priority attention in future campaigns.

Additional pets within households are likely to result from unplanned litters that could not be rehomed or from owners adopting friendly free-roaming dogs despite limited financial capacity to provide veterinary care. Since owned dogs account for the majority of dog bites in Sri Lanka, vaccination of owned dogs should receive equal priority to that of free-roaming dogs in efforts aimed at eliminating dog-mediated human rabies.

The authors recognize the compassionate attitudes of owners who adopt multiple animals and propose that vulnerable communities should receive home visits and additional clinic sessions in areas with poor performance.

### Incidence of TVT and skin infections

The increasing number of skin treatment cases probably reflects greater community reporting rather than increased dog density, and therefore does not provide reliable evidence of the success of the dog population management program. Nevertheless, the trend suggests increasing public awareness of animal welfare when quality veterinary services are provided free of charge.

Public awareness of TVT and skin diseases such as mange is likely to be low among the general public in Sri Lanka, and reliance on home remedies remains common. Positive word-of-mouth communication, reinforced by successful recoveries among friends' and neighbors’ pets, encourages owners to seek veterinary care for their own animals and to report affected free-roaming dogs to veterinary teams. Thus, improved access to veterinary care may enhance public participation in animal welfare activities.

### Program economics and economic returns

The economic analysis relied primarily on national estimates because regional data were unavailable. Nevertheless, the findings indicate that dog sterilization and vaccination generate economic benefits compared with the overall costs of rabies. Furthermore, accommodation costs could be largely eliminated in the long-term if government veterinarians and dedicated regional dog-catching teams were able to operate at a similar capacity.

### Plans for continuation

The perceived success of the program is reflected in the Provincial Council's decision to expand coverage to the entire North Central Province beginning in 2026. Although a different veterinary team will be responsible for vaccination and sterilization, the authors intend to remain involved in surveillance.

## STUDY LIMITATIONS

Several limitations should be considered when interpreting the findings of this study. First, the research team lacked sufficient financial and human resources to conduct systematic community dog surveys, which would have provided a more direct assessment of the intervention's impact on the overall dog population. Furthermore, age-related data were not stratified by sex, preventing a more detailed estimation of juvenile and adult survival rates for males and females. Such information would have enabled the derivation of more robust estimates regarding the proportion of the population requiring annual sterilization. Existing population models primarily rely on the proportion of sexually inactive females to estimate population growth rates. However, in a setting where a considerable proportion of owned males remain unsterilized, the inability to quantify the contribution of these males to impregnating free-roaming females represents an important knowledge gap that could not be addressed in the present study.

The use of clinical intake records and field captures as proxies for population trends may also be regarded as a limitation. Nevertheless, the cumulative numbers obtained correspond closely with the estimated regional dog population, suggesting that these measures provide useful information for monitoring real-world CNVR interventions in resource-limited settings, where funding constraints necessitate balancing research objectives with operational priorities. Similarly, the questionnaire survey may have been affected by selection bias because pet owners attending the clinics are likely to represent a more responsible segment of the community. However, substantial heterogeneity was observed across locations and responses regarding vaccination and sterilization coverage, and the 66.57% response rate is considered high by established standards, supporting the reliability of the findings.

Another major limitation was the lack of comprehensive and well-documented data on dog bites, human post-exposure prophylaxis, and suspected or laboratory-confirmed rabies cases within the project area. This deficiency reflects limitations of the national health information system and is beyond the authors' control. Although district-level data from the Ministry of Health were available and cited, these records do not necessarily reflect the specific epidemiological situation within the project's geographical boundaries. Consequently, the direct contribution of the intervention to reductions in dog bites, human post-exposure prophylaxis requirements, and rabies incidence could not be quantified accurately.

## CONCLUSION

This study represents the longest (>5 years) continuous CNVR intervention reported from a religious city in Asia and provides one of the few evaluations simultaneously encompassing owned and free-roaming dog populations, both sexes, and surrounding peri-urban communities. Although no significant reductions were observed in overall effort-adjusted dog counts, the absence of population growth over five years itself indicates substantial control of a population that would otherwise be expected to increase under tropical conditions. Male dog counts declined significantly by 9.7% annually, while owned female counts increased by 24.1%, accompanied by a shift in the overall male:female ratio from 1:2.1 to 1:3.6, suggesting increasing acceptance and adoption of female dogs. The intervention achieved high vaccination coverage among owned dogs, with 94% and 83% of dogs within and outside the municipality vaccinated at least once and 74% and 69%, respectively, receiving regular annual ARV. More than 60% of adult owned dogs and >78% of adult females had been sterilized. Spatial analyses further revealed previously undescribed sex-specific effects of the buffer zone, with each additional dog sterilized in the peripheral region reducing Zone 1 male catches and Zone 3 female catches, supporting the concept of “cutting down the supply and sealing the boundary.”

From a practical perspective, the findings demonstrate that sustained CNVR programs, integrated with community engagement and repeated vaccination campaigns, can achieve vaccination levels that approach or exceed the World Health Organization target for herd immunity, even in low-income and culturally complex environments. The study highlights the importance of addressing owned and free-roaming dogs simultaneously, prioritizing underserved neighborhoods, and strengthening legal frameworks governing sterilization, breeder registration, and responsible ownership. The estimated benefit-cost ratio of 1.5 further indicates that dog sterilization and vaccination are economically justified compared with the societal burden imposed by rabies.

A major strength of this study lies in its long duration, broad geographical coverage, inclusion of buffer zone strategies, and integration of demographic, spatial, behavioral, and economic analyses. The use of DAGs to justify analytical approaches and the identification of high-priority locations with poor sterilization performance provide additional translational value for future intervention planning.

Future studies should incorporate systematic dog population surveys, sex-specific demographic analyses, movement ecology, and longitudinal surveillance of dog bites, human post-exposure prophylaxis, and laboratory-confirmed rabies cases. Particular attention should be given to understanding the contribution of intact owned males to reproduction among free-roaming females and to elucidating the behavioral mechanisms underlying the sex-specific effects of buffer zones.

In conclusion, this study demonstrates that a sustained One Health-based CNVR approach, supported by community participation and strategic expansion beyond administrative boundaries, can stabilize dog populations, improve animal welfare, enhance responsible ownership, and maintain vaccination coverage consistent with rabies elimination goals. The findings introduce the buffer zone concept as a promising addition to domestic dog population management and provide a practical framework that may be adapted for other rabies-endemic regions seeking to achieve the target of zero human dog-mediated rabies deaths.

## RECOMMENDATIONS AND FUTURE DIRECTIONS

Based on the findings of this study, several recommendations can be proposed to strengthen future CNVR interventions and support the elimination of dog-mediated rabies.

Local government authorities should conduct baseline and follow-up dog population surveys before and during CNVR programs, incorporating age- and sex-specific information to facilitate evidence-based planning, monitoring, and evaluation. Annual surveys would enable a more accurate assessment of intervention outcomes and population dynamics. High-risk neighborhoods should be identified early and prioritized throughout the intervention period.

Urban areas should be divided into distinct operational zones according to anthropogenic characteristics, such as commercial districts, residential areas, and public spaces. Particular attention should be paid to ecological niches that may act as source or sink populations, such as cemeteries and abandoned areas, because these locations can substantially influence intervention outcomes.

The findings support establishing peripheral buffer zones around target areas. A minimum buffer radius of 3 km is recommended, although the optimal extent should be determined according to the roaming behavior and ecology of local dog populations. Because effective buffer implementation frequently extends beyond administrative boundaries, close collaboration among neighboring local authorities is essential.

To ensure equitable access to veterinary services, sterilization centers should be rotated among zones between campaigns. Animal welfare organizations implementing CNVR programs should actively collaborate with local authorities, community leaders, and religious institutions to improve logistics, community outreach, and identification of vulnerable areas.

As catchability declines over time, with the most approachable dogs being sterilized first, the success of long-term interventions increasingly depends on the expertise of the catching teams [25, 38]. Governments should therefore invest in recruiting and training professional dog catchers and establish certification programs. Maintaining dedicated regional catching teams would also reduce accommodation costs, which constitute a substantial proportion of operational expenditure.

Community participation should be strengthened through accessible reporting systems that allow residents to identify streets with high concentrations of free-roaming dogs. Voluntary dog feeders possess valuable local knowledge regarding dog movements and previously uncaptured individuals and should therefore be regarded as important stakeholders. Special approaches, including baiting and trapping, should be employed when dealing with stray and feral dogs that cannot be captured using conventional methods.

Public awareness campaigns should combine traditional and digital media, ranging from posters and handbills to social media platforms, to maximize community participation. Responsible pet ownership should be promoted from an early age through child-friendly educational activities. Stronger regulations governing dog breeding and sales are also necessary. Registration of breeders should be mandatory, and sterilization should be compulsory for all owned dogs that are not maintained by licensed breeders, thereby reducing the number of unwanted puppies entering the free-roaming population.

The findings of this study emphasize that sterilization programs should target both male and female dogs. Although many existing programs prioritize females, sexually intact males contribute substantially to population growth, nuisance behaviors, territorial aggression, and roaming. Awareness campaigns should particularly target first time owners and emphasize the importance of early age sterilization.

National governments should assume the leading role in mobilizing financial and human resources for large-scale CNVR programs while collaborating with animal welfare organizations and veterinary researchers. In addition to operational expenses, adequate research funding should be allocated for long-term (>5 years) surveillance, monitoring, and evaluation.

Future research should focus on long-term studies of sex-specific migration patterns, breeding behavior, and population dynamics of free-roaming dogs to improve understanding of the effects of mass sterilization programs. Further investigations should also explore owner demographics in high-risk areas and identify socioeconomic, logistical, and behavioral factors associated with poor vaccination and sterilization coverage. Such information will be essential for designing targeted interventions and accelerating progress toward the goal of zero human dog-mediated rabies deaths.

## DATA AVAILABILITY

The supplementary data can be made available from the corresponding author upon request.

## GENERATIVE AI DECLARATION

The authors declare that generative artificial intelligence (AI) tools were used solely to improve language, grammar, and readability during manuscript preparation. All scientific content, data analysis, interpretation of results, and conclusions were developed and verified by the authors. The authors take full responsibility for the accuracy, integrity, and originality of the work presented, and no AI tool was listed as an author.

## AUTHORS’ CONTRIBUTIONS

CN: Conceptualized the design of the dog population management program underlying this study, supervised data collection, and oversaw overall project administration. UAW: Oversaw adherence to surgical sterilization protocols and was responsible for program logistics and budgeting. AJ: Designed the questionnaire survey, conducted the literature review, performed data analysis and visualization, prepared geographic information system maps, and drafted the original manuscript. All authors have read and approved the final version of the manuscript.
